# Exploring the Ligand Chemical Space of the [Cr(Tpm^R^)_2_]^2+^ Spin-Crossover Family

**DOI:** 10.1021/acs.inorgchem.6c02008

**Published:** 2026-08-08

**Authors:** Arnau Garcia-Duran, Paula Pro, Jordi Cirera

**Affiliations:** Departament de Química Inorgànica i Orgànica and Institut de Recerca de Química Teòrica i Computacional, 16724 Universitat de Barcelona, Diagonal 645, 08028 Barcelona, Spain

## Abstract

The electronic and steric factors that control the spin-crossover behavior in the [Cr­(Tpm)_2_]^2+^ family (Tpm = tris­(pyrazolyl)­methane) have been investigated by means of density functional theory calculations. Systematic functionalization of the ligand framework reveals that both electronic and steric effects strongly influence the spin-state energetics and the corresponding transition temperatures. Substitution at different positions of the pyrazolyl rings affects the ligand field through distinct pathways, including resonance-mediated electronic effects and steric modulation of the metal–ligand bond lengths. The analysis of the electronic structure and ligand-field splitting through the relevant d-based molecular orbitals provides a molecular-level understanding of the observed trends and identifies the key factors in controlling the stability of the low- and high-spin states. These results allow for the construction of structure–property relationships within the [Cr­(Tpm)_2_]^2+^ family and provide guidelines for the rational design of new Cr­(II)-based spin-crossover compounds.

## Graphical abstract

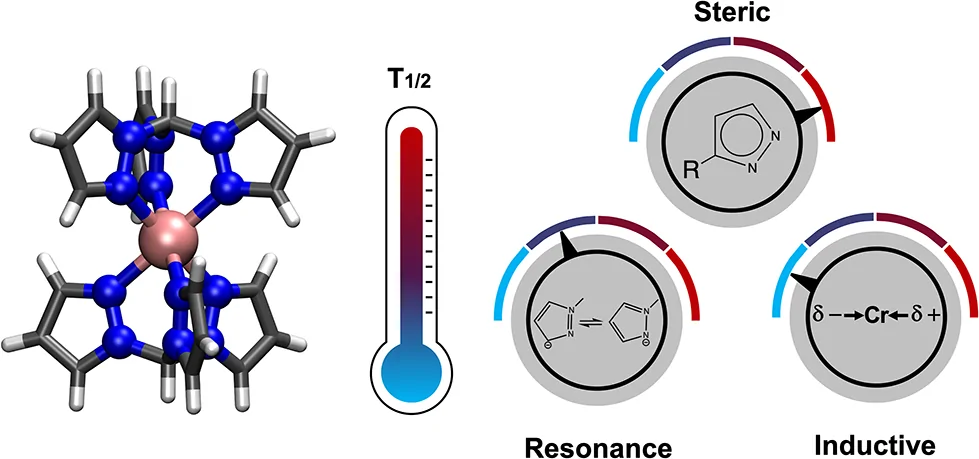

## 1 Introduction

Spin-crossover (SCO) molecular materials are characterized by the presence of two electronic states close in energy, resulting in bistable behavior associated with transitions between the low-spin (LS) and high-spin (HS) states.^1^ When thermally induced, the particular temperature at which the system has equal populations of both spin-sates is defined as the transition temperature (*T*_1/2_),^2^ which is a key parameter to control for actual technological implementations. These phenomena, first reported by Cambi and co-workers almost a century ago,^3^ are triggered by an external stimulus, being temperature the most common one, but can also be induced by pressure, electromagnetic radiation, or even guest molecules in supramolecular SCO systems.^4–8^ With the spin-state change, strong changes in the physical properties of the material (magnetic, optical, structural, etc.) can be observed, which makes this particular group of bistable materials quite appealing as potential molecular level switches for nanotechnological applications.^9–12^ The SCO field is overwhelmingly dominated by Fe­(II) coordination compounds, and multiple examples of such systems exhibiting SCO behavior have been reported in the literature for mononuclear, polynuclear, and extended 2*D*/3D systems, including MOF-type materials.^13–18^

Although in principle, spin-crossover could be possible for d^4^ to d^7^ metal ions, the SCO behavior of ions other than d^6^-Fe­(II) has been significantly less explored.^19,20^ In particular, Cr­(II)-based spin-crossover systems are quite scarce, and only few types of such systems have been reported in the literature. The first Cr­(II) SCO active complex was reported in 1989 by Halepoto and co-workers. The trans-[Cr­(depe)_2_I_2_] (depe = 1,2-bis­(diethylphosphino)­ethane) undergoes an abrupt transition with a *T*_1/2_ = 171 K.^21,22^ Later, In 1994, Hughes and co-workers synthesized and characterized the first examples of a dinuclear Cr­(II) SCO compound, the triple-decker organometallic complex [Cr_2_(μ:^2^:η^5^-P_5_)­(η^5^-C_5_Me_5_)_2_]­X (X = PF_6_^–^ or SbF_6_^–^), with very low transition temperatures (*T*_1/2_ ≈33 K for PF_6_^–^, *T*_1/2_ ≈23 K for SbF_6_^–^).^23^ In 1997, Sitzmann and co-workers studied the [Cr­(C_5_^i^Pr_4_)_2_] chromocene, which undergoes a transition at *T*_1/2_ ≈190 K,^24^ and in 2020, Becker and co-workers reported the Cr­(III)-based complex [Cr­(ddpd)_2_]^3+^ (ddpd = *N*,*N*′-dimethyl-*N*,*N*′-dipyridine-2-yl-pyridine-2,6-diamine), that can be reduced to its Cr­(II) analogue, which exhibits a reversible SCO transition that occurs gradually at room temperature.^25^ But perhaps the family that better illustrate the complexity of controlling the *T*_1/2_ in these systems was reported between 2006 and 2008 by Meredith and co-workers.^26,27^ The [Cr­(indenyl)_2_] family shows modulation of the *T*_1/2_ as a function of the ligand orientation but also as a function of the number of methyl groups inserted in the indenyl ligand, but more importantly, on the position of where these methyl groups are added. These systems have been intensively studied using computational tools,^28^ outlining models that can explain the observed trends in terms of the ligand orientation and functionalization, as well as the position of the methyl substituents in the indenyl ligand. Finally, in 2025, a new addition to the Cr­(II)-based SCO system was reported. Liu and co-workers reported the [Cr­(Tpm)_2_]­X_2_ complexes (Tpm = trispyrazoyl methane, X = BF_4_^–^ or ClO_4_^–^) exhibit SCO behavior with transition temperatures of 190 and 194 K depending on the counterion, while the closely related system [Cr­(Tpm^3,5‑Me^)_2_]­X_2_ shows either incomplete SCO with BF_4_^–^, or a fully high-spin state when ClO_4_^–^ is used as a counterion.^29^

It is clear thus that expanding the set of d^4^-Cr­(II)-based SCO systems is a challenging field. Thus, the computational study of these systems can shed light on how to explore the ligand chemical space to tune their *T*_1/2_ based on the effect that such modifications have over the metal–ligand field. In this work, electronic structure calculations on the [Cr­(Tpm^R^)_2_]^2+^ molecule have been carried out to analyze the role of steric, inductive, and resonant effects and their interplay over the splitting of the d-based molecular orbitals and thus the spin-crossover behavior of different members of this family.

## 2 Computational Details

All calculations have been done using the Orca6.0 electronic structure suite,^30^ with the r2SCAN^31,32^ functional with the def2TZVP^33,34^ basis set on all atoms and the DEFGRID3 integration grid. All energy optimizations were converged to 10^–8^ (TightSCF). The corresponding vibrational analysis was done on all optimized structures to compute the corresponding thermochemical data and ensure that they are global minimums along the potential energy surface. Enthalpic and entropic contributions were evaluated at 298.15 K using the harmonic approximation as implemented in the Orca6.0 package. Through this work, Δ*E* corresponds to the electronic energy difference between the high-spin and low-spin states, Δ*H* includes the corresponding thermal and zero-point corrections obtained from the vibrational analysis, and Δ*S* is the entropy difference between both states. Although the calculations were performed on isolated molecules, additional tests including explicit counterions indicate that the absolute *T*_1/2_ values can be affected by the crystal environment, whereas the electronic trends induced by ligand substitution remain preserved (see S9 in Supporting Information). n-Electron valence perturbation theory (NEVPT2) calculations were performed using the same software.^35^ In these calculations, we employed the def2TZVPP basis set, including the corresponding auxiliary basis set for the correlation and Coulomb fitting. The active space contains the 5 d-orbitals of the metal and 4 electrons, and the ab initio ligand-field theory (AILFT) approach was employed to extract the related orbitals.^36^ State-averaged CASSCF calculations were performed including all electronic roots arising from the CAS­(4,5) active space for the both spin multiplicities. The resulting AILFT orbital energies were subsequently used to evaluate the ligand-field splitting Δ_Oh_. In the present work, Δ_Oh_ is defined as the energy separation between the t_2g_-like and e_g_-like d-orbital manifolds obtained from the low-spin NEVPT2/AILFT calculations. We used all possible spin multiplicities and roots. Isocontours (0.03 e·Å^–3^) for the d-based molecular orbitals extracted from these calculations have been plotted using the VMD software,^37^ and the complete sets of energies and calculated Δ_Oh_ are provided in Table S2 in the Supporting Information.

## 3 Results

The calculation of spin-state energy gaps is, in general, a complex task. Previous work has shown that the TPSSh is, in principle, the proper method of choice because it provides with reasonable results for all metal ions from d^4^ to d^7^.^38^ Although this is true, other methods can be more quantitative toward *T*_1/2_ for specific metal ions. For instance, the B3LYP* functional has been shown to be more quantitative toward this quantity for Fe­(III) metal centers.^39,40^ For that reason, we tested different methods to compute *T*_1/2_ for the [Cr­(Tpm^H^)_2_] system (see S1 in Supporting Information) with an experimentally reported value of *T*_1/2_ = 190 or 194 K, using [BF_4_]^−^ or ClO_4_^–^ as counterions. While TPSSh still provides a reasonable result for the transition temperature (computed *T*_1/2_ = 396 K), across the other tested methods, the r2SCAN functional, which was already tested with good results for spin-state energy gaps in SCO systems,^41^ is the one that provides us with the closest value to the experimental one. The computed transition temperature, *T*_1/2_ = 224 K, is in excellent agreement with the experimentally reported values.^29^ For that reason, the r2SCAN functional was selected to study the [Cr­(Tpm^R^)_2_] family of SCO systems.

The parent complex, [Cr­(Tpm^H^)_2_]^2+^, exhibits SCO behavior, while [Cr­(Tpm^3,5‑Me^)_2_]^2+^ is a high-spin molecule. This effect indicates that ligand functionalization can modify the SCO properties of [Cr­(Tpm)_2_]^2+^ systems. For that reason, we decided to study how ligand modifications can affect the *T*_1/2_ in such a family of compounds. In particular, the functionalization of the positions 3, 4, and 5 of each pyrazole ring (see Figure 1) in the tris­(pyrazolyl)­methane ligand was modified to evaluate how the interplay between steric, inductive, and resonant effects and how such modifications can alter the electronic structure of the different members of this family. The apical position of the ligand has also been modeled as carbon or boron (see Figure 1). The substituents were chosen to span a broad range of electron-donating, electron-withdrawing, and steric effects while maintaining a computationally accessible data set.

**Figure 1:**
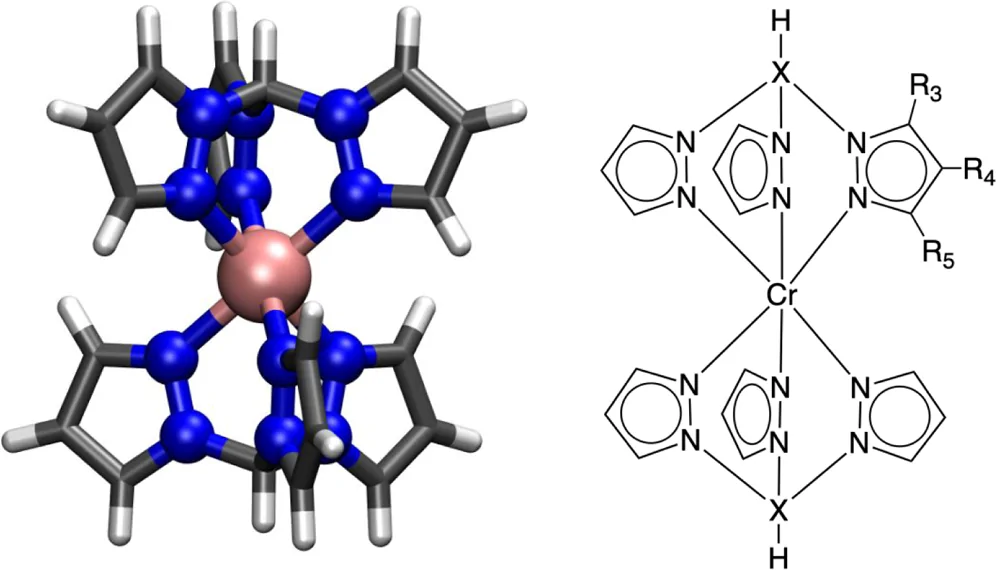
Left, molecular representation of the [Cr­(Tpm^H^)_2_]^2+^ system (blue for nitrogen, gray for carbon, white for hydrogen, and pink for chromium). Right, schematic depiction of the studied system with functionalization in positions R_3_, R_4_, and R_5_ (substitution takes place in all pyrazole rings) as well as the apical position of the ligand (X = C or B).

### 3.1 [Cr­(Tpm^3,5^‑^R^)_2_] System

As indicated above, a significant difference in the SCO properties of the [Cr­(Tpm)_2_]^2+^ systems has been experimentally observed when comparing the [Cr­(Tpm)_2_]^2+^ with the [Cr­(Tpm^3,5‑Me^)_2_]^2+^ molecule. Experimentally, the first one is a SCO compound (*T*_1/2_ = 190 or 194 K depending on the counterion), while the latter is a high-spin system. Thus, our first test was to check if this behavior was properly reproduced by the calculations. Indeed, while the computed *T*_1/2_ for the [Cr­(Tpm)_2_]^2+^ indicates SCO behavior, with a positive Δ*H* = 2.04 kcal/mol, the computed spin-state energy gap for the [Cr­(Tpm^3,5‑Me^)_2_]^2+^ is already negative (Δ*H* = −0.43 kcal/mol), correctly reproducing the high-spin ground-state nature of this compound. Given the clear influence that substituents in these positions seems to have, we analyzed the effect of other substituents in the same positions ([Cr­(Tpm^3,5‑R^)_2_]^2+^, see Table 1). This data indicates that strong inductive effects expected from the 3,5 functionalization of the pyrazole ring are quickly counterbalanced by the steric congestion of the R_5_ position.

**Table 1:** Spin-State Energy Difference, Enthalpy Difference, Entropy Difference, and Computed Transition Temperature for the [Cr­(Tpm^3,5‑R^)_2_]^2+^ Systems, as Well as the Steric Parameter for the Ligand (r_L_Tpm). Energies and Enthalpies in kcal·mol^–1^, Entropies in cal·K^–1^·mol^–1^, Temperatures in K, and r_L_Tpm in Angstroms^a^

-R_3_,R_5_	r_L_Tpm	Δ*E*	Δ*H*	Δ*S*	*T*_1/2_
-H	1.20	1.27	2.05	9.13	224
-F	1.46	–0.49	–0.63	7.73	–81
-Cl	1.82	0.53	0.48	7.94	61
-Br	1.86	0.66	0.54	8.33	65
-I	2.04	1.61	1.43	10.24	140
-NH_2_	2.22	–0.47	–0.46	6.41	–71
-CH_3_	2.27	–0.30	–0.43	6.39	–67
-CF_3_	2.79	–2.93	–2.73	8.83	–309

a Negative values of *T*_1/2_ indicated that the ground state is the high-spin state. R_3_ and R_5_ denote the substituent located at the corresponding position of the pyrazole rings as shown in Figure 1.

To quantify the steric effect, we modeled the substituents as spheres and define their radii using the covalent and vdW radii reported by Alvarez.^42,43^ This parameter (r_L_Tpm) is taken as the vdW radii for atomic substituents, and for molecular ones is defined as the sum of the covalent radii of the involved species plus the vdW radii of the furthest atomic substituent. According to this, the r_L_Tpm­(H) is 1.20 Å, which is its vdW radii for H, but for –CH_3_ is the sum of the covalent radii of C and H (0.76 + 0.31), plus the vdW radii of hydrogen (1.20), adding to a total of 2.27 Å.

It is worth mentioning that for r_L_Tpm bigger than 2.1 Å, the steric effects gain relevance and hence suppress the transition. Meanwhile, if the r_L_Tpm is small enough, the transition can occur and be affected by the inductive and resonant effects. For those cases, the computed transition temperature shows the expected tendency with the electronegativity of the substitution (see Figure 2).

**Figure 2:**
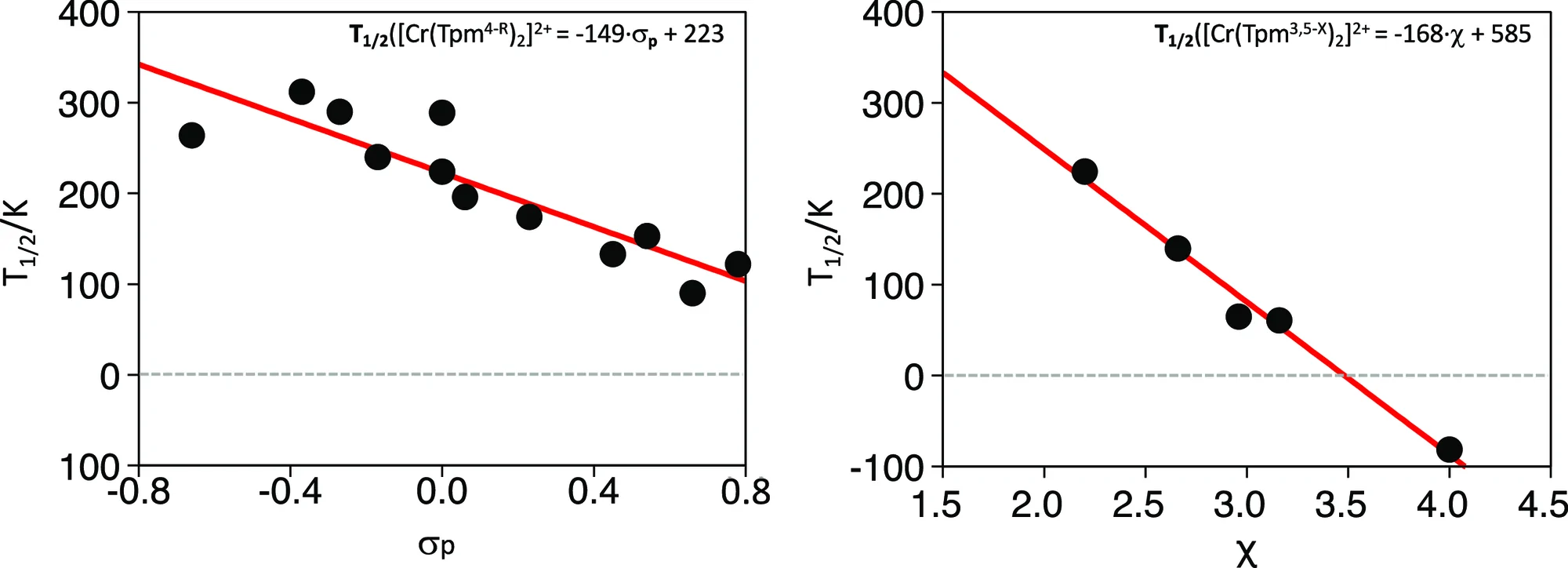
Left: Linear fit for the computed *T*_1/2_ vs the σ_p_ Hammett constant for the [Cr­(Tpm^4‑R^)_2_]^2+^ family of compounds (R = NH_2_, OH, OCH_3_, CH_3_, SMe, H, F, Cl, COOH, CF_3_, CN, NO_2_, R^2^ = 0.80). Right: Linear fit for the computed *T*_1/2_ vs the χ for the [Cr­(Tpm^3,5‑X^)_2_]^2+^ family of compounds (X = F, Cl, Br, I, and H, *R* ^2^ = 0.97).

### 3.2 [Cr­(Tpm^3‑R^)_2_] Systems and [Cr­(Tpm^5‑R^)_2_] Systems

To understand the role of the substitution in each of the two positions: either –R_3_ or –R_5_, independent studies on ligand modification in each position have been done. We started by modifying the –R_3_ position in the [Cr­(Tpm^3‑R^)_2_]^2+^ system. The results are summarized in Table 2.

**Table 2:** Spin-State Energy Difference, Enthalpy Difference, Entropy Difference, and Computed Transition Temperature for the [Cr­(Tpm^3‑R^)_2_]^2+^ Systems, as Well as the Resonant Descriptor R_*x*_. Energies and Enthalpies in kcal·mol^–1^, Entropies in cal·K^–1^·mol^–1^, and Temperatures in K^a^

-R_3_	R_*x*_	Δ*E*	Δ*H*	Δ*S*	*T*_1/2_
-NH_2_	–0.0412	–0.23	0.07	6.51	11
-SMe	–0.0193	1.91	1.88	6.82	276
-F	–0.0173	–0.45	–0.34	7.84	–43
-CH_3_	–0.0106	2.43	2.75	8.17	336
-Cl	–0.0041	0.93	1.05	7.94	132
-H	0.0000	1.27	2.05	9.13	224
-CF_3_	0.0125	2.69	3.22	6.65	484
-CN	0.0147	2.03	2.65	8.20	324
-COOH	0.0217	3.69	4.29	6.45	665
-NO_2_	0.0253	3.26	4.27	5.98	715

a Negative values of *T*_1/2_ indicated that the ground state is the high-spin state. R_3_ denotes the substituent located at the corresponding position of the pyrazole rings as shown in Figure 1.

Functionalization of the R_3_ position (Table 2) shows a general increase in *T*_1/2_ as the substituents become more electron withdrawing (EWG). Although this trend can be qualitatively captured by the Hammett σ_p_ constants (see S5 in Supporting Information), the correlation is not sufficiently strong to support a purely resonant or inductive interpretation. Therefore, we searched for descriptors capable of splitting the different electronic effects involved. Given the remote position of this substitution, this behavior should be consistent with a modulation of the ligand π-system character, with resonance-mediated electronic effects likely contributing to the observed increase in ligand field strength. Thus, we decided to use the newly introduced descriptors for inductive and resonant effects, namely, I_*x*_ and R_*x*_, which provides with a robust framework for quantifying substituent effects in aromatic systems.^44^ For the R_3_ functionalization, a significantly stronger correlation between the spin-state energy gap (or the computed *T*_1/2_) with the R_*x*_ descriptor was found, indicating that resonance effects provide a more consistent explanation of the observed trends (see S5 in Supporting Information). This suggests that electronic communication through the conjugated ligand framework plays a more important role than purely inductive effects in determining the ligand-field strength and, consequently, the spin-crossover properties of these complexes.

Single functionalization of the R_5_ position in the [Cr­(Tpm^5‑R^)_2_] systems leads once again to a strong steric congestion that pulls the ligands apart, moving rapidly toward a high-spin state as the size of the substituent increases. In fact, the numbers of reported in Table 3 are quite similar to the ones reported for the [Cr­(Tpm^3,5‑R^)_2_] systems (Table 1), thus indicating the extreme sensitivity of the R_5_ position toward steric effects.

**Table 3:** Spin-State Energy Difference, Enthalpy Difference, Entropy Difference, and Computed Transition Temperature for the [Cr­(Tpm^5‑R^)_2_]^2+^ Systems, as Well as the Steric Parameter for the Ligand (r_L_Tpm)^a^

-R_5_	r_L_Tpm	Δ*E*	Δ*H*	Δ*S*	*T*_1/2_
-H	1.20	1.27	2.05	9.13	224
-Cl	1.82	0.78	0.61	9.25	66
-NH_2_	2.22	–0.27	–0.10	4.12	–23
-CH_3_	2.27	–1.27	–1.38	8.48	–163
-CF_3_	2.79	–2.94	–2.84	10.48	–271

a Energies and enthalpies in kcal·mol^–1^, entropies in cal·K^–1^·mol^–1^, temperatures in K, and r_L_Tpm in Angstroms. Negative values of *T*_1/2_ indicated that the ground state is the high-spin state. R_5_ denotes the substituent located at the corresponding position of the pyrazole rings as shown in Figure 1.

### 3.3 [Cr­(Tpm^4‑R^)_2_] Systems

Since the R_5_ position seems to have a strong sterical contribution that leads toward the high-spin state, we focus our attention on the R_4_ position, which should have strong resonance effects on the pyrazole ligand and minimize, if any, the steric effect. Results for functionalization of the [Cr­(Tpm^4‑R^)_2_]^2+^ system are reported in Table 4. As can be seen from Figure 2, a clear correlation appears between the σ_p_ Hammett constant,^45^ which quantifies the electron-donating or electron-withdrawing effect of a substituent (in the para position), and the computed *T*_1/2_, thus indicating that both resonant and inductive effects are at play in controlling the ligand ability to tune the SCO properties when the ligand is functionalized in the R_4_ position. A close inspection to the data shows also a good correlation of the computed *T*_1/2_ with the resonant R_*x*_ parameter, which points out a higher weight of such effects in controlling the transition temperature in such systems. More importantly, if we attempt to build a similar trend with the inductive parameter I_*x*_, no correlation is observed (see S3 in Supporting Information).

**Table 4:** Spin-State Energy Difference, Enthalpy Difference, Entropy Difference, and Computed Transition Temperature for the [Cr­(Tpm^4‑R^)_2_]^2+^ Systems, as Well as the σ_p_ Hammett Para Constant^a^

-R_4_	σ_p_	Δ*E*	Δ*H*	Δ*S*	*T*_1/2_
-NH_2_	–0.66	1.22	1.72	6.53	264
–OH	–0.37	1.62	2.34	7.51	312
-OCH_3_	–0.27	1.85	2.23	7.69	290
-CH_3_	–0.17	1.64	2.15	8.95	240
-SMe	0.00	1.77	2.45	8.50	289
-H	0.00	1.27	2.05	9.13	224
-F	0.06	1.44	1.73	8.84	196
-Cl	0.23	1.27	1.55	8.91	174
-COOH	0.45	0.87	1.18	8.84	133
-CF_3_	0.54	1.09	1.39	9.12	153
-CN	0.66	–0.04	0.76	8.45	90
-NO_2_	0.78	0.78	1.06	8.69	122

a Energies and enthalpies in kcal·mol^–1^, entropies in cal·K^–1^·mol^–1^, temperatures in K. R_4_ denotes the substituent located at the corresponding position of the pyrazole rings as shown in Figure 1.

### 3.4 Effect of the Apical Atom in the [Cr­(TpX^H^)_2_]^*n*+^

The tris­(pyrazolyl)­methane (Tpm) ligand possesses a closely related analogue, tris­(pyrazolyl)­borate (Tp^–^), in which the apical carbon center is replaced by boron and the ligand becomes formally anionic. Since many transition-metal complexes with the Tp^–^ ligands have been reported, we also investigated the influence of this modification on the spin-crossover properties. Our calculations show that upon replacement of carbon by the boron group, the spin-state energy gap is almost twice as big (2.05 vs 4.73 kcal/mol, Tpm and Tp^–^, respectively). This translates into a significantly larger value for the transition temperature in the [Cr­(Tp^H^)_2_] system, around 456 K, while the [Cr­(Tpm^H^)_2_]^2+^ has been computed at 224 K. Therefore, one should expect the [Cr­(Tp^H^)_2_] system to be a low-spin Cr­(II)-based molecule. Because of the anionic nature of the Tp^–^ ligand, electron density is more effectively transmitted to the pyrazolyl donor atoms, strengthening the ligand field at the Cr­(II) center and increasing the d-orbital splitting. Consequently, the low-spin configuration is stabilized, relative to the high-spin state. However, as also happens in the Tpm series, steric congestion can be used to reduce the ligand-field strength. Indeed, the calculated spin-state energy gap of [Cr­(Tp^3,5–CH3^)_2_] falls within the range expected for spin crossover, yielding a predicted *T*_1/2_ value of 83 K.

## 4 Discussion

Results from the previous section seem to indicate that functionalization in different positions of the Tpm ligand leads to a clear competition between inductive, resonant, and steric effects. While inductive effects can affect the ligand field around the Cr­(II) metal ion in a subtle way, steric ones will lead, in general, toward the high-spin situation. This effect is observed experimentally when comparing the [Cr­(Tpm^3,5,H^)_2_]^2+^ and [Cr­(Tpm^3,5‑Me^)_2_]^2+^ systems (Table 1). Thus, we will start discussing this steric effect, because it is limiting in terms of having the possibility of generating SCO behavior in such molecules. Results from Table 1 and Table 3 shown a clear correlation between the size of the substituent in the –R_5_ position and the spin-state energy gap (or computed *T*_1/2_). In particular, and from the data analysis, it seems that any substituent in that position with an r_L_Tpm larger than 2.10 Å will lead to a high-spin situation. This effect can be clearly quantified in terms of the relevant d-based MOs using the ab initio ligand field theory (AILFT) approach to analyze the output from NEVPT2 calculations on the low-spin-optimized geometries for the [Cr­(Tpm^3,5,H^)_2_]^2+^ and [Cr­(Tpm^3,5‑Me^)_2_]^2+^ systems (see Figure 3). The enlargement of the Cr–N bond lengths observed in the low-spin [Cr­(Tpm^3,5‑Me^)_2_]^2+^, with an average Cr–N = 2.08 Å bond length, compared with the value for the low-spin [Cr­(Tpm^3,5,H^)_2_]^2+^ (Cr–N = 2.04 Å) indicates that the steric congestion pushes the ligands far away from the Cr­(II) center, thus lowering the antibonding interactions with the d_*xz*_ and d_*yz*_ orbitals, which in turn decreases their energy and reduces the effective Δ_Oh_ from 18085 cm^–1^ to 16669 cm^–1^ ([Cr­(Tpm^3,5‑H^)_2_]^2+^ vs [Cr­(Tpm^3,5‑Me^)_2_]^2+^, see Figure 4).

**Figure 3:**
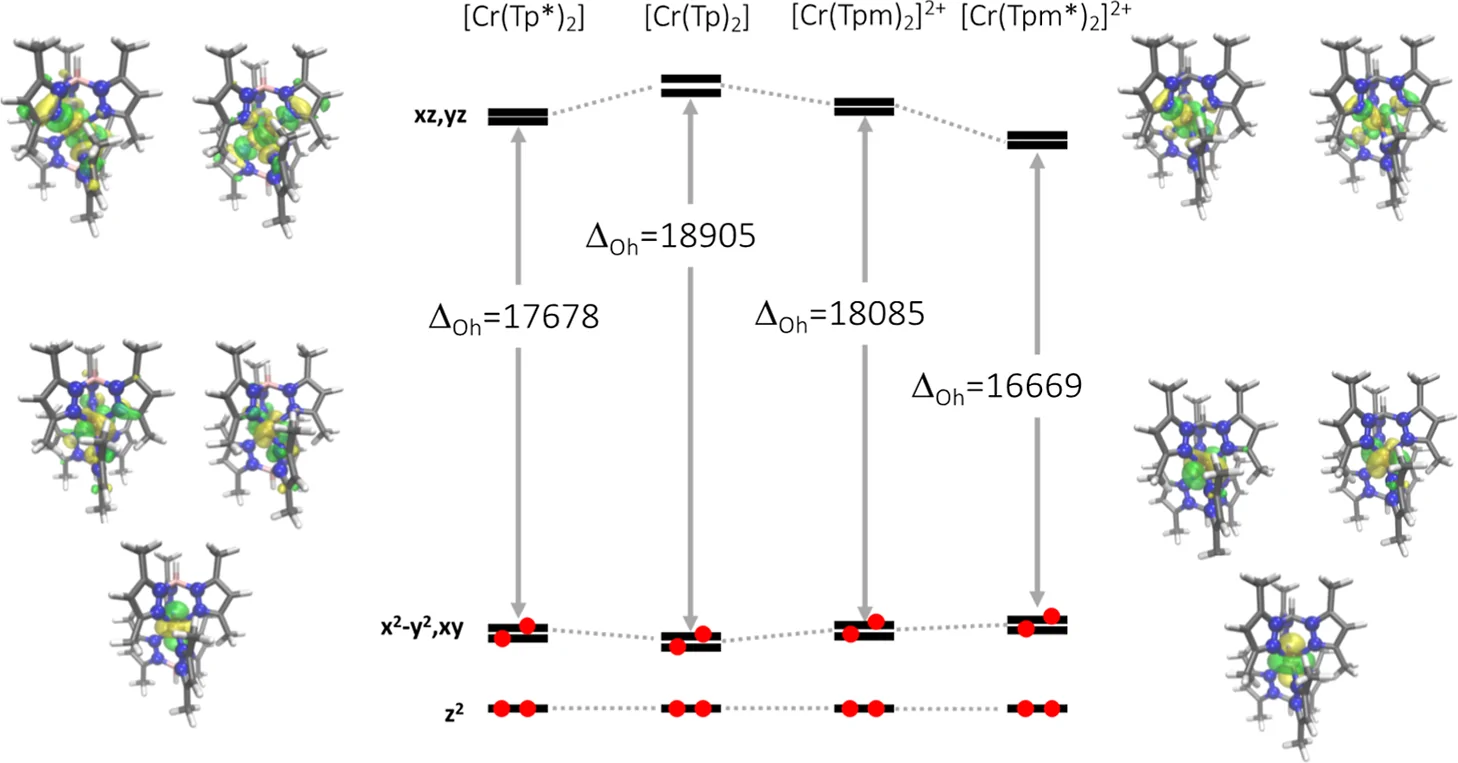
d-Based MO diagram, including the computed Δ_Oh_ for each system (in cm^–1^), for [Cr­(Tp^3,5‑H^)_2_] (left isosurfaces), [Cr­(Tp^3,5–Me^)_2_], ([Cr­(Tpm^3,5‑H^)_2_]^2+^, and [Cr­(Tpm^3,5‑Me^)_2_]^2+^ (right isosurfaces).

**Figure 4:**
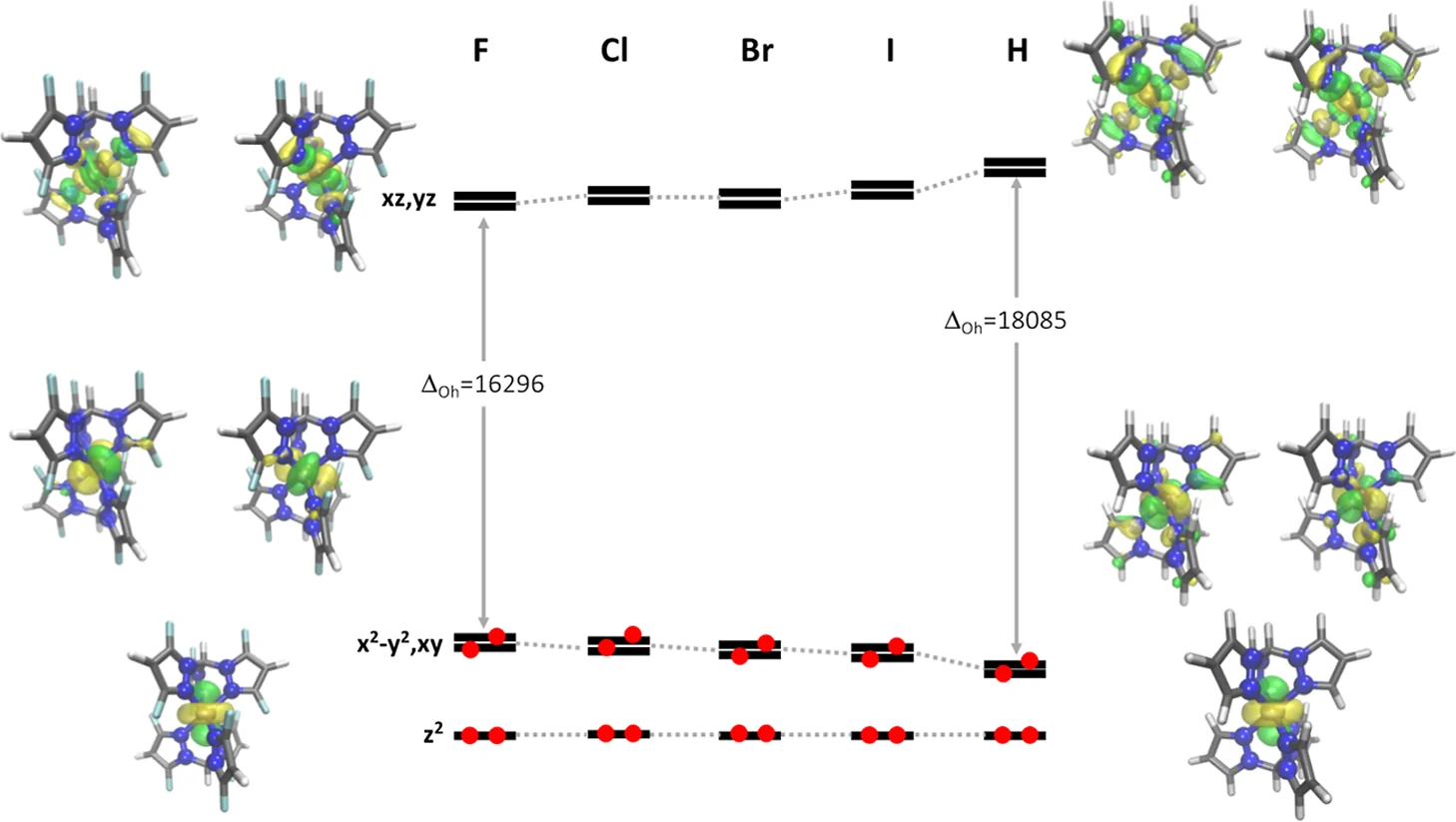
d-Based MO diagram, including the Δ_Oh_ for the limit cases (in cm^–1^), for the [Cr­(Tpm^3,5‑X^)_2_]^2+^. From left to right, X = F (left isosurfaces), Cl, Br, I, and H (right isosurfaces).

Replacement of the apical carbon atom by boron involves the change of the neutral tris­(pyrazolyl)­methane ligand (Tpm) to the formally anionic tris­(pyrazolyl)­borate ligand (Tp^–^). Consistent with the stronger donor character of Tp^–^,^46,47^ the AILFT calculations reveal an increase in the ligand-field splitting from 18085 cm^–1^ for the Tpm complex to 18905 cm^–1^ for the corresponding Tp^–^ analogue. This increase in Δ_Oh_ is accompanied by a larger spin-state energy gap and a substantially higher calculated *T*_1/2_ value, indicating stronger stabilization of the low-spin state. Additionally, in Tp^–^, the boron atom is less electronegative than the carbon one in Tpm, meaning that the pyrazole rings in Tp^–^ are more electron-rich, which enhances their σ-donor ability toward the metal. All of this translates in a marked preference for the low-spin state. However, the steric congestion effect is also clearly visible for the [Cr­(Tp)_2_] systems with the [Cr­(Tp^3,5–Me^)_2_], with a Δ_Oh_ = 17678 cm^–1^. Thus, it is important to notice that, as observed for the Tpm ligand, addition of methyl groups in the R_5_ position of the Tp^–^ one lowers the computed *T*_1/2,_ setting its value in the right window for SCO to occur.

Although functionalization of the R_5_ position generally favors the high-spin state because of the steric congestion introduced by bulky substituents, the use of small substituents minimizes these steric effects, allowing the electronic contribution of the substituent to become the dominant effect. This behavior is nicely illustrated by the [Cr­(Tpm^3,5‑X^)_2_]^2+^ family (X = F, Cl, Br, or I), in which the halogen atoms never cross the 2.1 Å r_L_Tpm limit. As can be seen, the average Cr–N bond lengths in the low-spin state remain nearly constant (around 2.07 Å) across the series, indicating that geometric distortions are minimal. Within this subset, increasing substituent electronegativity systematically lowers both Δ_Oh_ and *T*_1/2_. In particular, fluorine, the most electronegative substituent, gives rise to the smallest Δ_Oh_ value by lowering the energy of the Cr–N σ-antibonding d-MO orbitals (Figure 4), thus stabilizing the high-spin state. On the other hand, less electronegative halogens progressively increase Δ_Oh_, leading to a corresponding increase in the spin-state energy gap and *T*_1/2_.

From the above, it is clear that inductive and resonant effects can play a major role in tuning *T*_1/2_ when decoupled from steric ones. These effects can be much better observed when functionalization of the –R_3_ and –R_4_ positions by itself is done (Tables 2 and 4). The R_4_ substituent influences the electronic structure of the pyrazole ligand predominantly through resonant effects. This is nicely reflected by the correlation between the σ_p_ Hammett constant and the computed *T*_1/2_. Stronger electron-donating groups (EDGs), with negative σ_p_ Hammett constants, increase the electron density on the *N*-donor atom, enhancing the Cr–N σ interaction, which in turns raises the energy of the antibonding orbitals. This effect can be seen in both, the d-based MO diagram (Figure 5) as well as in the shortening of the average Cr–N bond length (see S8 in Supporting Information). The opposite is observed for electron-withdrawing groups (EWGs), with positive σ_p_ Hammett constants,^45^ that reduce the electron density at the *N*-donor atom leading to, ultimately, smaller Δ_Oh_. To compare, the Δ_Oh_ for [Cr­(Tpm^4‑Me^)_2_]^2+^ and [Cr­(Tpm^4‑CF3^)_2_]^2+^ are 19262 and 18933 cm^–1^, respectively, right above and below the Δ_Oh_ for [Cr­(Tpm)_2_]^2+^ parent compound (Δ_Oh_ = 19056 cm^–1^) (see Figure 5). The relevance of the resonant effects in this position can also be quantified using the R_*X*_ values, recently developed,^44^ which show a correlation with only such parameter but not with their inductive counterpart (I_*x*_) (see S3 in Supporting Information). Thus, the absence of a significant correlation with inductive descriptors, together with the excellent correlations obtained for σ_p_ and R_*x*_, indicates that the substituent effect is transmitted predominantly through resonance rather than through inductive σ-polarization effects.

**Figure 5:**
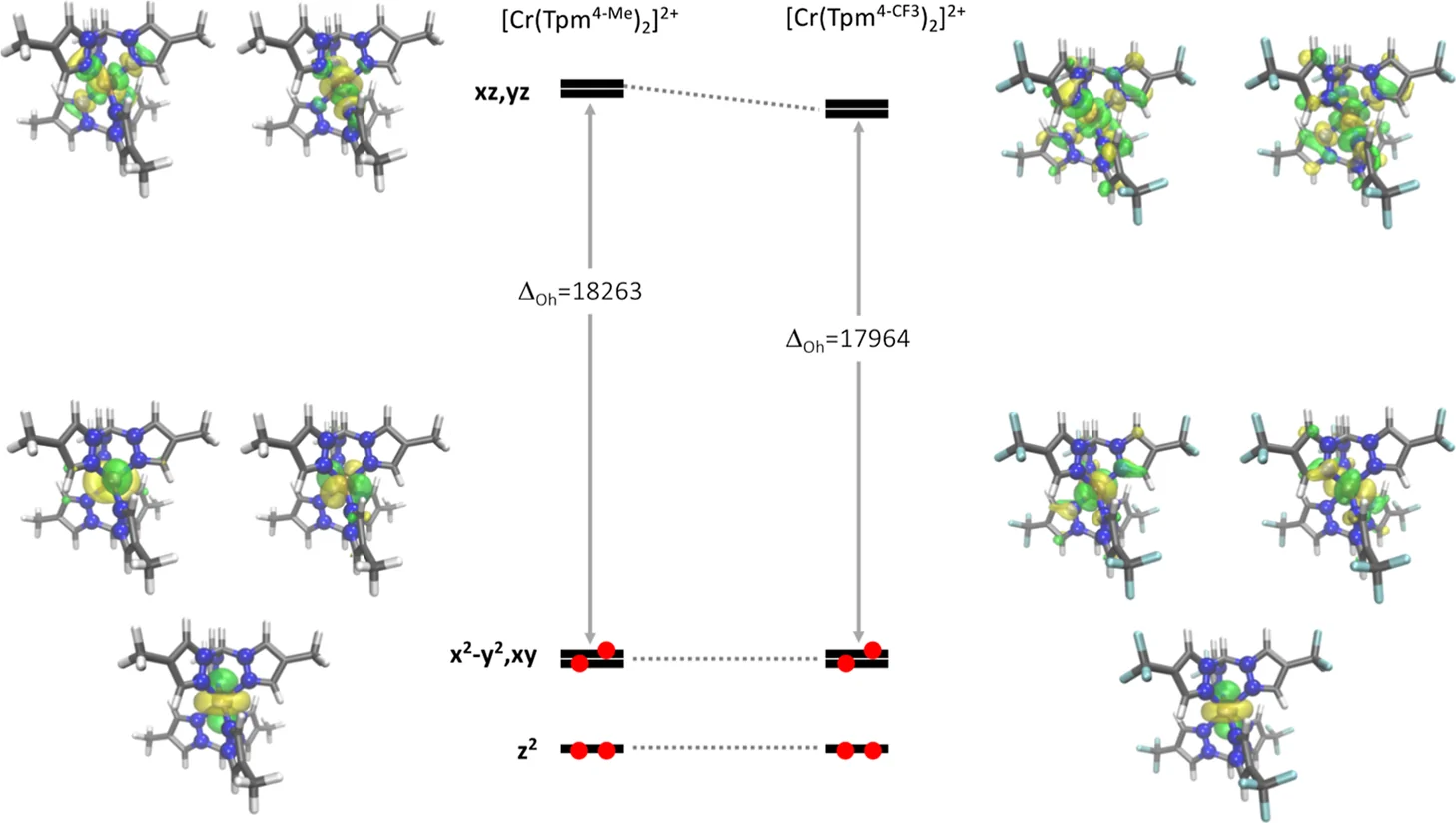
Schematic comparison of the relevant d-based MO diagrams, including the computed Δ_Oh_ for each system (in cm^–1^), for the [Cr­(Tpm^4‑CH3^)_2_]^2+^ (left isosurfaces) and [Cr­(Tpm^4‑CF3^)_2_]^2+^ (right isosurfaces) systems.

Finally, functionalization of the R_3_ position is expected to produce only minor steric perturbations around the Cr­(II) center due to its relatively remote location within the ligand structure. Instead, substituent effects should be transmitted primarily through electronic communication along the pyrazole-based conjugated system. Former studies in the closely similar [Fe­(Tpm^3‑CH3^)_2_] system shows that functionalization of this position leads to the reverse effect as observed for the R_5_ position, that is, the spin-state energy gap opens, reaching higher *T*_1/2_.^48,49^ The authors propose that repulsion of the methyl groups in the R_3_ position closes the ligand bite angle, stabilizing short Fe–N bonds (i.e., the low-spin state). Even though our results reproduce the alignment of the methyl groups and the inverse trend in *T*_1/2_ when the R_3_ position is functionalized, no significant differences in the bite angle between the systems [Cr­(Tpm^3‑CH3^)_2_]^2+^, [Cr­(Tpm)_2_]^2+^, and [Cr­(Tpm^5‑CH3^)_2_]^2+^ are observed (see S4 in Supporting Information). Therefore, the observed trend seems not to be steric in origin but rather electronic. Although the trend can be qualitatively captured by the σ_p_ Hammett constant, the strongest correlation is obtained with the resonance descriptor R_*x*_ (see Table 2). This indicates that resonance-mediated electronic communication through the conjugated ligand framework provides with the most consistent explanation for the observed increase in Δ*E* and *T*_1/2_. Thus, EWG substituents increase the ligand-field splitting, stabilizing the low-spin state relative to the high-spin configuration and leading to a larger spin-state energy gap.

It is worth noticing that in all cases, a strong Jahn–Teller distortion is observed upon transition from the low-spin to the high-spin state, as can be seen analyzing the ratio between the longest and the shortest Cr–N bond (see S7 in the Supporting Information) and as observed experimentally.^29^ Importantly, across the methyl-substituted series, which allows us to compare functionalization in all the positions, the bond length ratio in the high-spin state oscillates between 1.15 and 1.18 regardless of substitution position, indicating that the substituents do not significantly alter the geometric character of the high-spin state. Therefore, the substituent effects on the spin-crossover properties are predominantly electronic in origin, modulating the ligand field and the spin-state energy gap rather than acting through changes in the magnitude of the Jahn–Teller distortion.

## 5 Conclusions

In this work, an extensive computational study of the [Cr­(Tpm^R^)_2_]^2+^ system spin-crossover properties has been outlined. The proposed computational method is able to quantitatively reproduce the experimentally available data,^29^ which allowed us to further explore the ligand chemical space. Our results outline a delicate balance between steric, resonant, and inductive effects that modulate the ligand field splitting of the Cr­(II) metal center. It is clear that the [Cr­(Tpm^5‑R^)_2_]^2+^ systems have a marked tendency to become high-spin molecules, due to the steric congestion of the substituents in the –R_5_ position, a trend also observed in the [Cr­(Tpm^3,5‑R^)_2_]^2+^ for the same reason. However, when small substituents are introduced at the R_5_ position, steric effects are largely minimized and the spin-state energetics become primarily governed by electronic effects. In the 3,5-halogenated series, increasing substituent electronegativity systematically lowers both Δ_Oh_ and *T*_1/2_, consistent with the observed modulation of the Cr–N σ-antibonding orbitals. Similarly, resonant effects in the [Cr­(Tpm^4‑R^)_2_]^2+^ systems dominate the value of *T*_1/2_, tuning its value between 90 and 310 K. Although the steric congestion of the –R_5_ position seems to lock the highs-spin state, this can be harnessed if the closely related ligand Tp^–^ is used instead of the Tpm one. The [Cr­(Tp)_2_] system has a large spin-state energy gap that leads to a low-spin situation. However, by adding methyl groups in the R_3_ and R_5_ positions, one can weaken the ligand field of the [Cr­(Tp^3,5–CH3^)_2_] system to generate a potential candidate to exhibit SCO. Overall, our results show how electronic structure calculations can help rationalize, in terms of the electronic structure of the system, the computed trends for all systems studied in this work, which paves the road for the design of new Cr­(II)-based spin-crossover systems by optimizing the interplay between steric and electronic effects over the spin-state energy gap.

## Supplementary Material



## Data Availability

A data set collection of computational results is available in the ioChem-BD repository and can be accessed via 10.19061/iochem-bd-6-646.

## Notes

Density functional approximation benchmark calculations; NEVPT2/AILFT d-orbital energy diagrams and molecular orbitals for representative complexes; correlations between the R_*x*_, I_*x*_, and Hammett descriptors and the computed spin-crossover temperatures; bite-angle, N–N distance, continuous shape measure, Jahn–Teller distortion, and Cr–N bond-length analyses for all studied systems; and effect of explicit BF_4_^–^ and ClO_4_^–^ counterions on the calculated transition temperatures (PDF)

## References

[1] Gütlich, P. ; Goodwin, H. A. Spin Crossover in Transition Metal Compounds III. Topics in Current Chemistry; Springer Berlin, Heidelberg, 2004; Vol. 268. 10.1007/b96439.

[2] Kahn, O. Molecular Magnetism; VCH, 1993.

[3] Cambi L., Szegö L. (1931). Über die magnetische Susceptibilität
der komplexen Verbindungen. Ber. Dtsch. Chem.
Ges..

[4] Darawsheh M., Barrios L. A., Roubeau O., Teat S. J., Aromí G. (2016). Guest-Light-
and Thermally-Modulated Spin Crossover in [Fe^II^
_2_] Supramolecular Helicates. Chem. Eur J..

[5] Gütlich P., Hauser A. (1990). Thermal and light-induced
spin crossover in iron­(II)
complexes. Coord. Chem. Rev..

[6] König E., Ritter G., Waigel J., Goodwin H. A. (1985). The effect
of pressure
on the thermal hysteresis of the first-order spin transition in bis­(1,10-phenanthroline-2-carbaldehyde
phenylhydrazone) iron (II) complexes. J. Chem.
Phys..

[7] McConnell A.
J., Aitchison C. M., Grommet A. B., Nitschke J. R. (2017). Subcomponent Exchange
Transforms an FeII4L4 Cage from High- to Low-Spin, Switching Guest
Release in a Two-Cage System. J. Am. Chem. Soc..

[8] Steinert M., Schneider B., Dechert S., Demeshko S., Meyer F. (2016). Spin-State
Versatility in a Series of Fe4 [2 × 2] Grid Complexes: Effects
of Counteranions, Lattice Solvent, and Intramolecular Cooperativity. Inorg. Chem..

[9] Halcrow, M. A. Spin-Crossover Materials Properties and Applications; Wiley, 2013.

[10] Kahn O., Kröber J., Jay C. (1992). Spin Transition Molecular Materials
for displays and data recording. Adv. Mater..

[11] Kamilya S., Dey B., Kaushik K., Shukla S., Mehta S., Mondal A. (2024). Realm of Spin
State Switching Materials: Toward Realization of Molecular and Nanoscale
Devices. Chem. Mater..

[12] Ridier K., Hoblos A., Calvez S., Lorenc M., Nicolazzi W., Cobo S., Salmon L., Routaboul L., Molnár G., Bousseksou A. (2025). Optical properties
and photonic applications
of molecular spin-crossover materials. Coord.
Chem. Rev..

[13] Halder G. J., Chapman K. W., Neville S. M., Moubaraki B., Murray K. S., Létard J.-F., Kepert C. J. (2008). Elucidating the
Mechanism of a Two-Step Spin Transition in a Nanoporous Metal–Organic
Framework. J. Am. Chem. Soc..

[14] Hogue R. W., Singh S., Brooker S. (2018). Spin crossover in discrete polynuclear
iron­(ii) complexes. Chem. Soc. Rev..

[15] Neville S. M., Halder G. J., Chapman K. W., Duriska M. B., Moubaraki B., Murray K. S., Kepert C. J. (2009). Guest Tunable
Structure and Spin
Crossover Properties in a Nanoporous Coordination Framework Material. J. Am. Chem. Soc..

[16] Neville S. M., Halder G. J., Chapman K. W., Duriska M. B., Southon P. D., Cashion J. D., Létard J.-F., Moubaraki B., Murray K. S., Kepert C. J. (2008). Single-Crystal to Single-Crystal
Structural Transformation and Photomagnetic Properties of a Porous
Iron­(II) Spin-Crossover Framework. J. Am. Chem.
Soc..

[17] Ohba M., Yoneda K., Agustí G., Muñoz M. C., Gaspar A. B., Real J. A., Yamasaki M., Ando H., Nakao Y., Sakaki S., Kitagawa S. (2009). Bidirectional
Chemo-Switching
of Spin State in a Microporous Framework. Angew.
Chem., Int. Ed..

[18] Southon P. D., Liu L., Fellows E. A., Price D. J., Halder G. J., Chapman K. W., Moubaraki B., Murray K. S., Létard J.-F., Kepert C. J. (2009). Dynamic Interplay between Spin-Crossover and Host–Guest
Function in a Nanoporous Metal–Organic Framework Material. J. Am. Chem. Soc..

[19] Harding D. J., Harding P., Phonsri W. (2016). Spin crossover in iron­(III) complexes. Coord. Chem. Rev..

[20] Olguín J. (2020). Unusual metal
centres/coordination spheres in spin crossover compounds. Coord. Chem. Rev..

[21] Halepoto D. M., Holt D. G. L., Larkworthy L. F., Leigh G. J., Povey D. C., Smith G. W. (1989). Spin crossover in
chromium­(II) complexes and the crystal
and molecular structure of the high spin form of bis­[1,2-bis­(diethylphosphino)­ethane]­di-iodochromium­(II). J. Chem. Soc., Chem. Commun..

[22] Halepoto D. M., Holt D. G. L., Larkworthy L. F., Povey D. C., Smith G. W., Leigh G. J. (1989). Spin crossover in chromium­(II) complexes. Polyhedron.

[23] Hughes A. K., Murphy V. J., O’Hare D. (1994). Synthesis,
X-ray structure and spin
crossover in the triple-decker complex [(η5-C5Me5)­Cr­(μ2:η5-P5)­Cr­(η5-C5Me5)]+[A]–(A
= PF6, SbF6). J. Chem. Soc., Chem. Commun..

[24] Sitzmann H., Schär M., Dormann E., Kelemen M. (1997). Octaisopropylchromocen:
Das erste Chromocen mit kontinuierlichem low spin/high spin-Übergang. Z. Anorg. Allg. Chem..

[25] Becker P. M., Forster C., Carrella L. M., Boden P., Hunger D., van Slageren J., Gerhards M., Rentschler E., Heinze K. (2020). Spin Crossover and Long-Lived Excited States in a Reduced
Molecular Ruby. Chem..

[26] Meredith M. B., Crisp J. A., Brady E. D., Hanusa T. P., Yee G. T., Brooks N. R., Kucera B. E., Young V. G. (2006). High-Spin
and Spin-Crossover
Behavior in Monomethylated Bis­(indenyl)­chromium­(II) Complexes. Organometallics.

[27] Meredith M. B., Crisp J. A., Brady E. D., Hanusa T. P., Yee G. T., Pink M., Brennessel W. W., Young V. G. (2008). Tunable Spin-Crossover Behavior in Polymethylated Bis­(indenyl)­chromium­(II)
Complexes: The Significance of Benzo-Ring Substitution. Organometallics.

[28] Navarro L., Rodriguez F., Cirera J. (2021). Controlling the spin-crossover behavior
of the [Cr­(indenyl)­2] family via ligand functionalization. Dalton Trans..

[29] Liu S., Liu Q., Deng Y.-F., Zhang Y.-Z. (2025). Trigonal antiprismatic mononuclear
Cr­(ii) spin-crossover complexes. Inorg. Chem.
Front..

[30] Neese F. (2022). Software update:
The ORCA program system—Version 5.0. WIREs Comput. Mol. Sci..

[31] Furness J. W., Kaplan A. D., Ning J., Perdew J. P., Sun J. (2020). Correction
to Accurate and Numerically Efficient r2SCAN Meta-Generalized Gradient
Approximation. J. Phys. Chem. Lett..

[32] Furness J. W., Kaplan A. D., Ning J., Perdew J. P., Sun J. (2020). Accurate and
Numerically Efficient r2SCAN Meta-Generalized Gradient Approximation. J. Phys. Chem. Lett..

[33] Weigend F. (2006). Accurate Coulomb-fitting
basis sets for H to Rn. Phys. Chem. Chem. Phys..

[34] Weigend F., Ahlrichs R. (2005). Balanced basis sets
of split valence, triple zeta valence
and quadruple zeta valence quality for H to Rn: Design and assessment
of accuracy. Phys. Chem. Chem. Phys..

[35] Angeli C., Cimiraglia R., Malrieu J.-P. (2001). N-electron valence state perturbation
theory: a fast implementation of the strongly contracted variant. Chem. Phys. Lett..

[36] Singh S. K., Eng J., Atanasov M., Neese F. (2017). Covalency
and chemical bonding in
transition metal complexes: An ab initio based ligand field perspective. Coord. Chem. Rev..

[37] Humphrey W., Dalke A., Schulten K. (1996). VMD: Visual
molecular dynamics. J. Mol. Graphics.

[38] Cirera J., Via-Nadal M., Ruiz E. (2018). Benchmarking Density Functional Methods
for Calculation of State Energies of First Row Spin-Crossover Molecules. Inorg. Chem..

[39] Vidal D., Cirera J., Ribas-Arino J. (2021). Accurate calculation
of spin-state
energy gaps in Fe­(III) spin-crossover systems using density functional
methods. Dalton Trans..

[40] Vidal D., Cirera J., Ribas-Arino J. (2023). Fine-tuning of the spin-crossover
properties of Fe­(III) complexes via ligand design. Phys. Chem. Chem. Phys..

[41] Mejía-Rodríguez D., Trickey S. B. (2020). Spin-Crossover from a Well-Behaved, Low-Cost meta-GGA
Density Functional. J. Phys. Chem. A.

[42] Alvarez S. (2013). A cartography
of the van der Waals territories. Dalton Trans..

[43] Cordero B., Gómez V., Platero-Prats A. E., Revés M., Echeverría J., Cremades E., Barragán F., Alvarez S. (2008). Covalent radii revisited. Dalton
Trans..

[44] Comas-Vila G., Salvador P. (2025). Capturing electronic
substituent effect with effective
atomic orbitals. Phys. Chem. Chem. Phys..

[45] Hansch C., Leo A., Taft R. W. (1991). A survey
of Hammett substituent constants and resonance
and field parameters. Chem. Rev..

[46] Kitajima, N. ; Tolman, W. B. Coordination Chemistry with Sterically Hindered Hydrotris­(pyrazolyl)­borate Ligands: Organometallic and Bioinorganic Perspectives. In Progress in Inorganic Chemistry; Wiley, 1995; pp 419–531.

[47] Trofimenko S. (1993). Recent advances
in poly­(pyrazolyl)­borate (scorpionate) chemistry. Chem. Rev..

[48] Goodman M. A., DeMarco M. J., Tarasek S. E., Nazarenko A. Y., Brennessel W., Goodman M. S. (2014). Iron complexes of
tris­(pyrazolyl)­ethane
ligands methylated in the 3-, 4-, and 5-positions. Inorg. Chim. Acta.

[49] Goodman M. A., Nazarenko A. Y., Casavant B. J., Li Z., Brennessel W. W., DeMarco M. J., Long G., Goodman M. S. (2012). Tris­(5-methylpyrazolyl)­methane:
Synthesis and Properties of Its Iron­(II) Complex. Inorg. Chem..

