# Supplementary material for: Exploring the Ligand Chemical Space of the [Cr(TpmR)2]2+ Spin-Crossover Family
Source: Inorg Chem. 2026 Aug 8;65(33):19267–74. doi: 10.1021/acs.inorgchem.6c02008 (PMC13508996; doi:10.1021/acs.inorgchem.6c02008)
Supplement: Supplementary file 1 [file ic6c02008_si_001.pdf]

Supporting Information

Exploring the Ligand Chemical Space of the $[Cr(Tpm^{R})_2]^{2+}$ Spin-Crossover family

*Arnau Garcia-Duran[1], Paula Pro[1] and Jordi Cirera[1]**

[1] Departament de Química Inorgànica i Orgànica and Institut de Recerca de Química Teòrica i Computacional, Universitat de Barcelona, Diagonal 645, 08028 Barcelona, Spain

e-mail: jordi.cirera@qi.ub.es

S1. Density Functional Approximations Benchmark S1

S2. NEVPT2/AILFT d-based MOs for selected systems S2

S3. R_x and I_x vs $T_{1/2}$ correlations for $[Cr(Tpm^{4\text{-}R})_2]^{2+}$ systems S7

S4. Bite angle and N-N distance for $[Cr(Tpm^{R\text{-}CH3})_2]^{2+}$ (R = 3 or 5) and $[Cr(Tpm)_2]^{2+}$ S8

S5. $[Cr(Tpm^{R3})_2]^{2+}$ correlations with σ_p S9

S6. CShM analysis for all computed systems in this work S11

S7. Jahn-Teller analysis of the studied systems S13

S8. Bond length analysis for all studied systems in this work S15

S9. Effect of counterion inclusion in $T_{1/2}$ calculation S17

S1. Benchmark of different exchange/correlation density functional approximations against the experimental value for the $[Cr(Tpm)_2]^{2+}$ system (experimental value $T_{1/2}$ = 190 K). Enthalpic and entropic contributions were evaluated at 298.15 K using conventional statistical thermodynamics within the ideal-gas, rigid-rotor, and harmonic-oscillator approximation as implemented in ORCA 6.0.

**Functional**	**ΔE**	**ΔH**	**ΔS**	$\mathbf{T_{1/2}}$
TPSSh	3.69	3.41	8.620	397
OPBE	-7.49	-7.32	9.156	-799
SCAN	6.21	6.40	11.001	582
r2SCAN	1.28	2.05	9.056	226
r2SCANh	-3.53	-3.61	8.352	-432
PBE0	-3.08	-4.19	5.098	-823

All energies and enthalpies in kcal·mol^{-1}, entropies in cal·K^{-1}·mol^{-1} and temperatures in K.

S2.d-based Molecular Orbitals for different ligands studied in this work.

Tpm<br>Δ_{Oh}=18085cm^{-1}	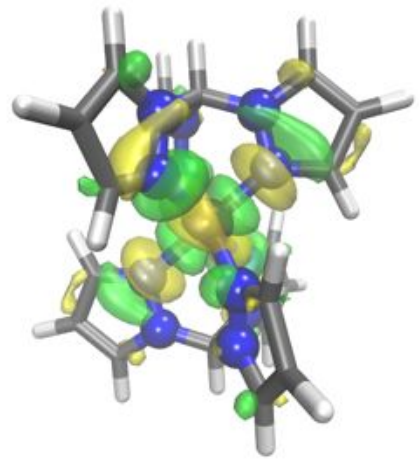<br>20973 cm^{-1}	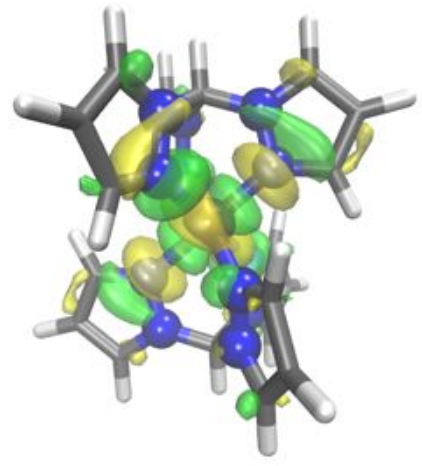<br>20880 cm^{-1}	
	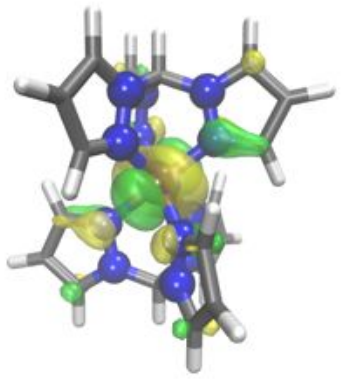<br>2795 cm^{-1}	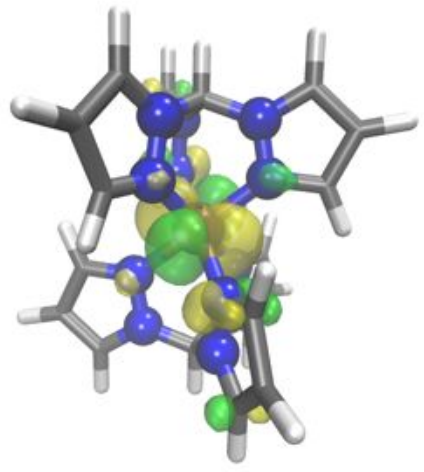<br>2762 cm^{-1}	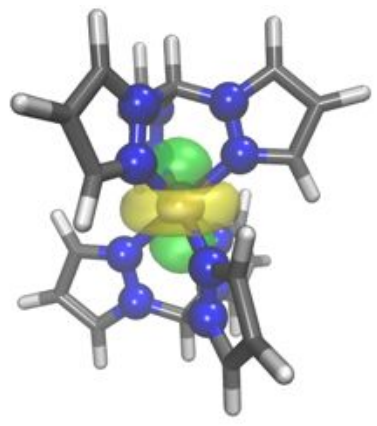<br>0 cm^{-1}

Tp^{-}<br>Δ_{Oh}=18905cm^{-1}	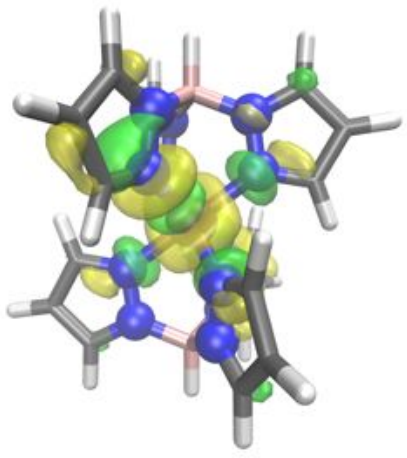<br>21790 cm^{-1}	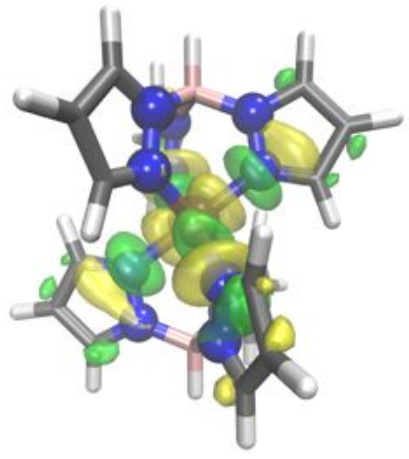<br>21269 cm^{-1}	
	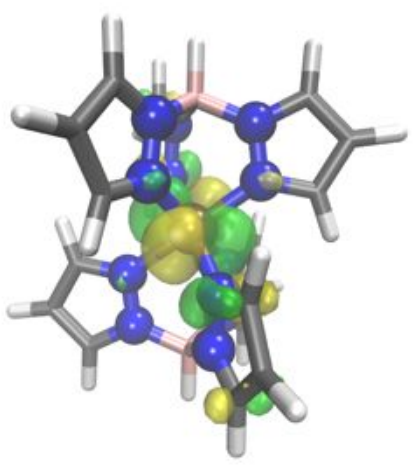<br>2364 cm^{-1}	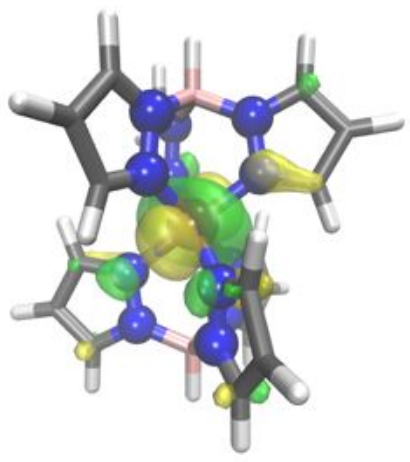<br>2262 cm^{-1}	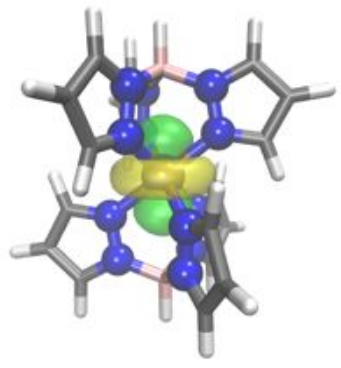<br>0 cm^{-1}

Tpm-3,5-Me

Δ_{Oh}=16669cm^{-1}

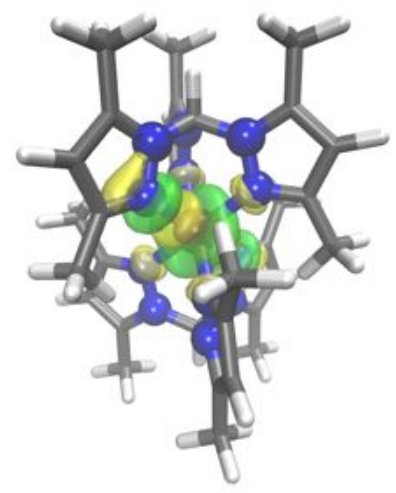

19725 cm^{-1}

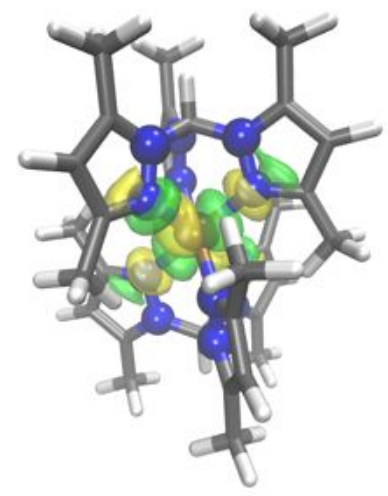

19626 cm^{-1}

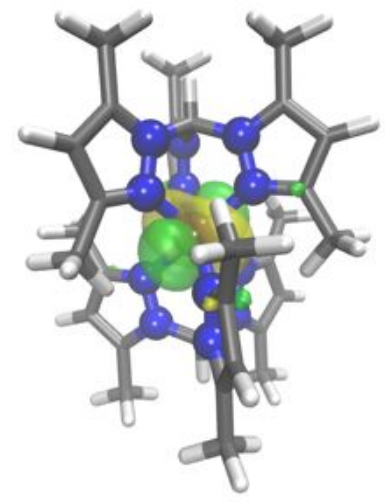

2957 cm^{-1}

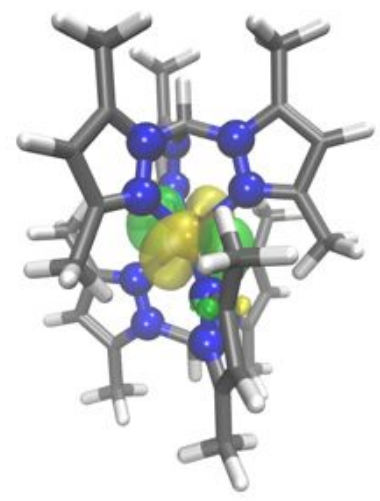

2947 cm^{-1}

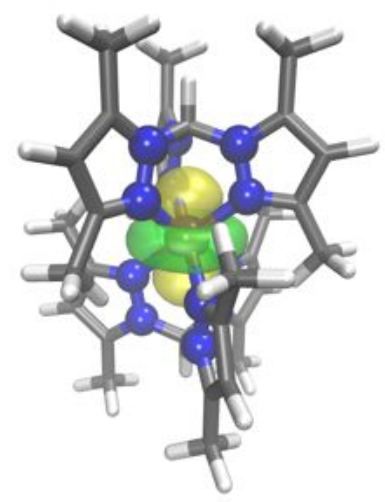

0 cm^{-1}

Tp-3,5-Me

Δ_{Oh}=17678cm^{-1}

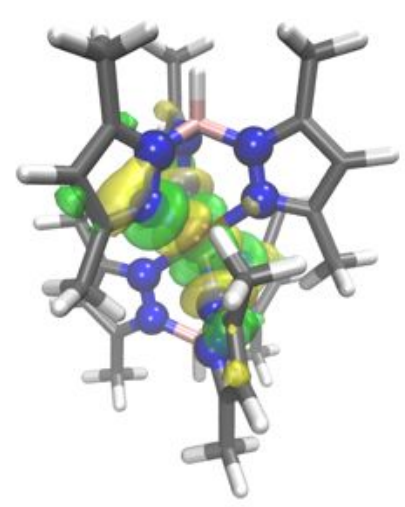

20579 cm^{-1}

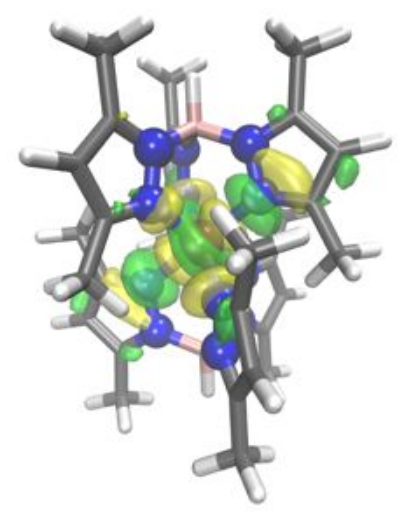

20328 cm^{-1}

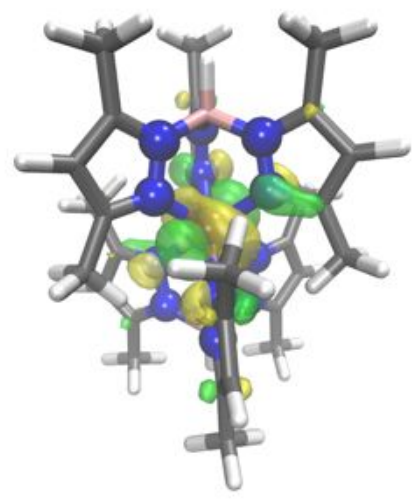

2650 cm^{-1}

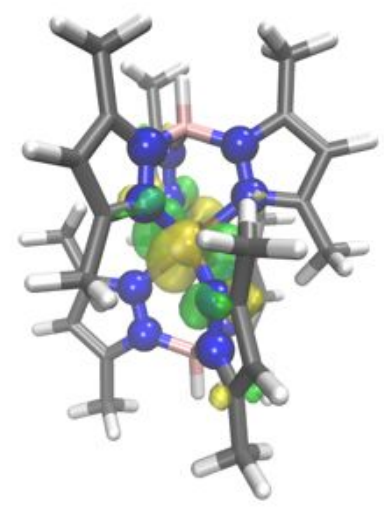

2583 cm^{-1}

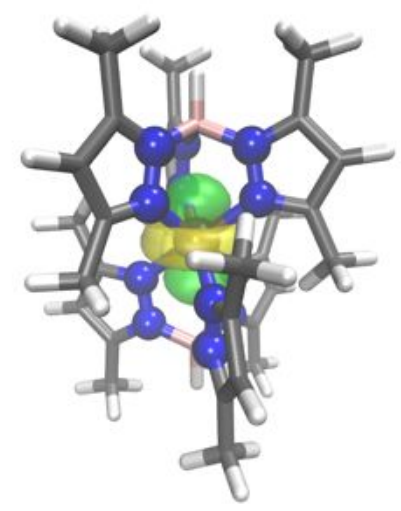

0 cm^{-1}

Tpm-4-Me<br>Δ_{Oh}=18263cm^{-1}	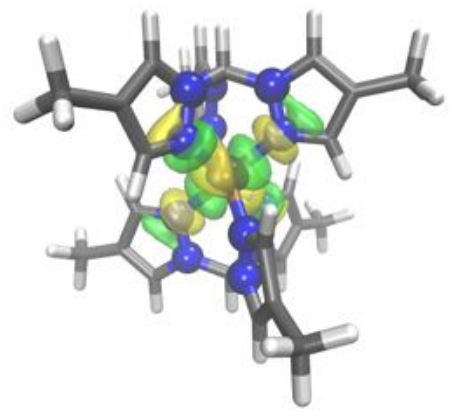<br>21160 cm^{-1}	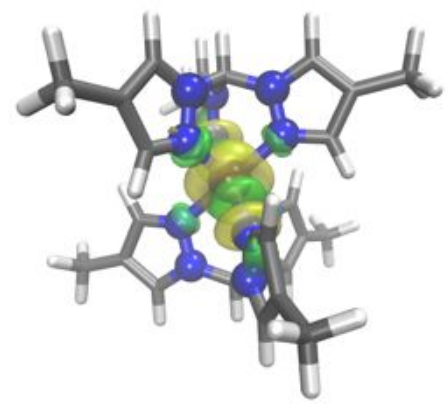<br>21016 cm^{-1}	
	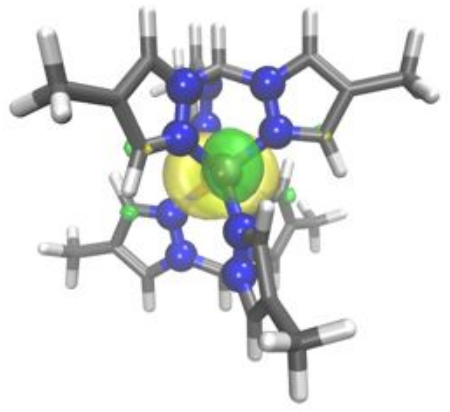<br>2753 cm^{-1}	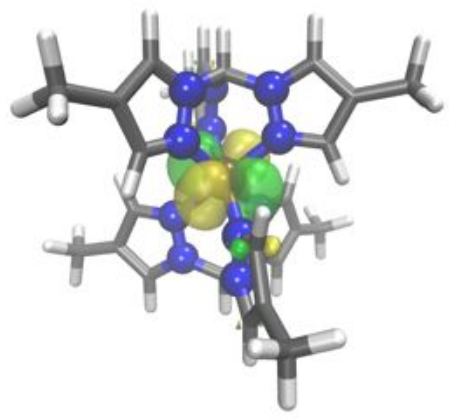<br>2726 cm^{-1}	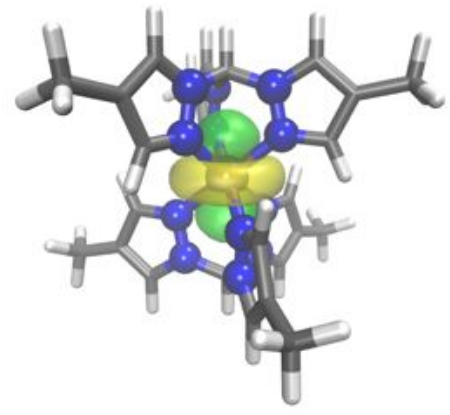<br>0 cm^{-1}

Tpm-4-CF_3<br>Δ_{Oh}=17964cm^{-1}	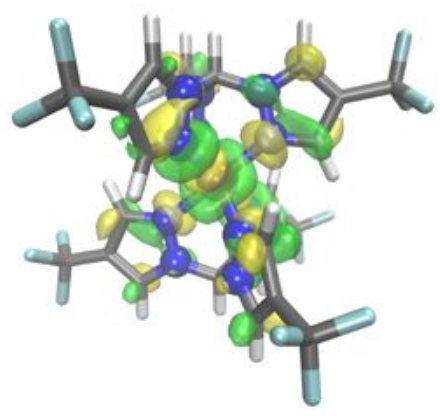<br>20802 cm^{-1}	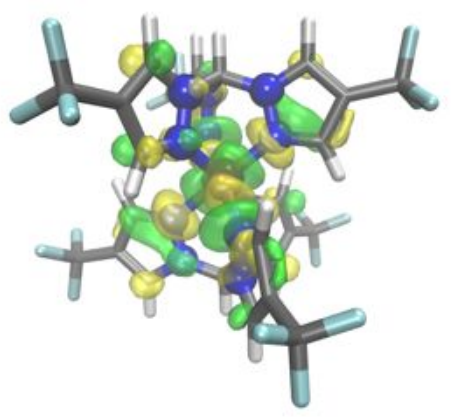<br>20741 cm^{-1}	
	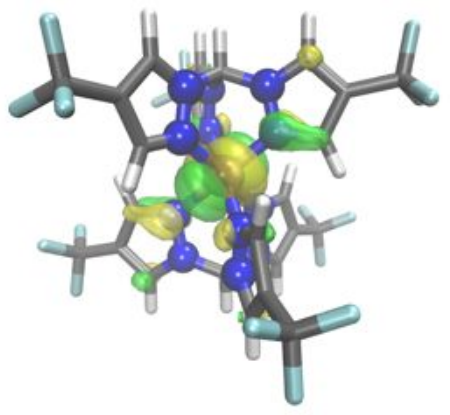<br>2777 cm^{-1}	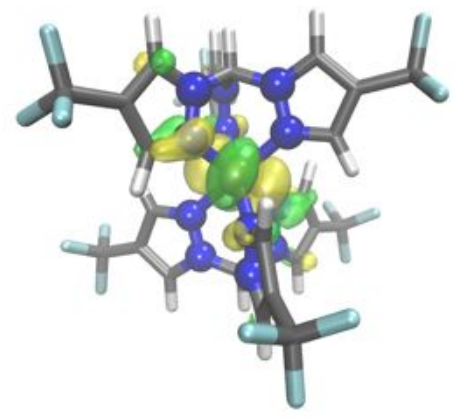<br>2738 cm^{-1}	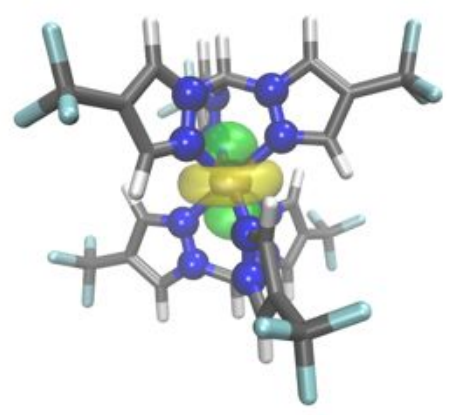<br>0 cm^{-1}

Tpm-3,5-F<br>Δ_{Oh}=16296cm^{-1}	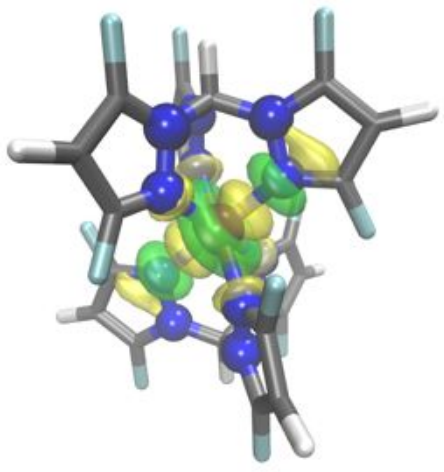<br>19669 cm^{-1}		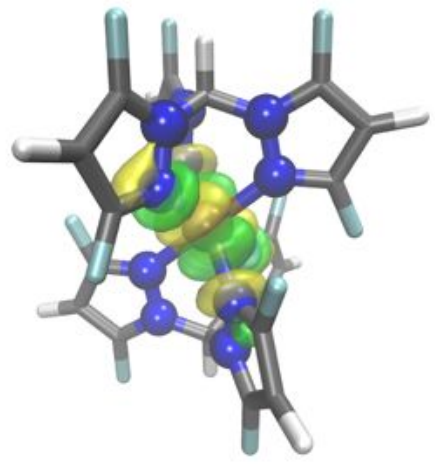<br>19652 cm^{-1}
	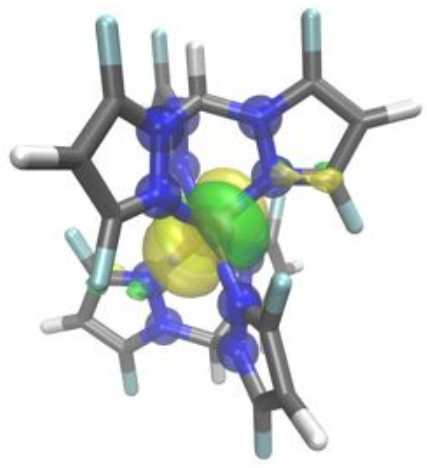<br>3356 cm^{-1}	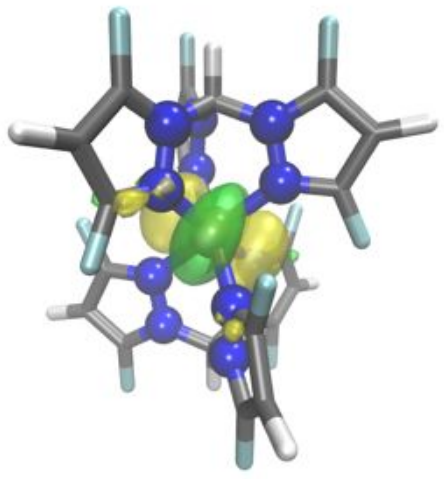<br>3354 cm^{-1}	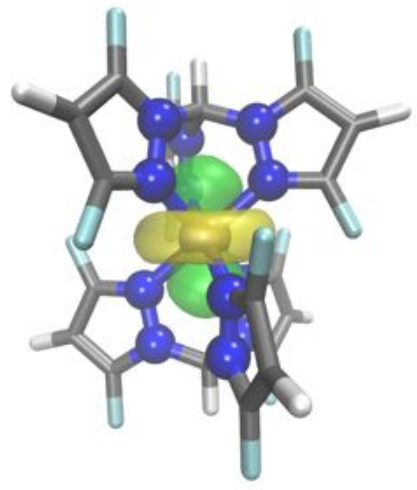<br>0 cm^{-1}

Tpm-3,5-Cl<br>Δ_{Oh}=16518cm^{-1}	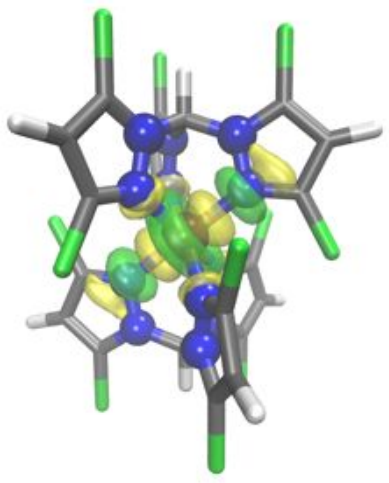<br>19808 cm^{-1}		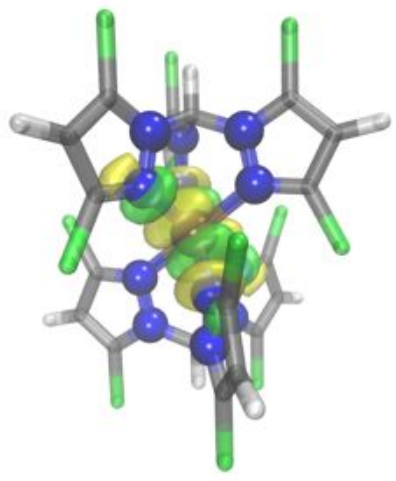<br>19761 cm^{-1}
	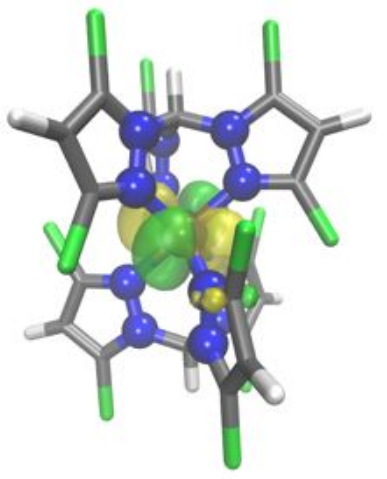<br>3243 cm^{-1}	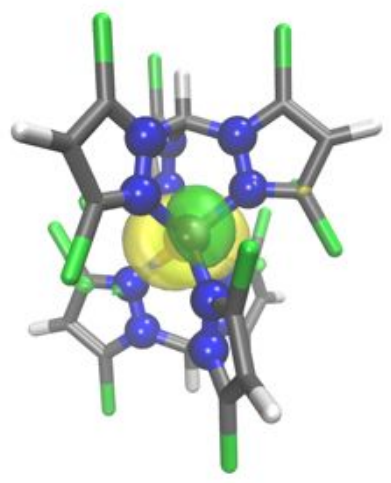<br>3240 cm^{-1}	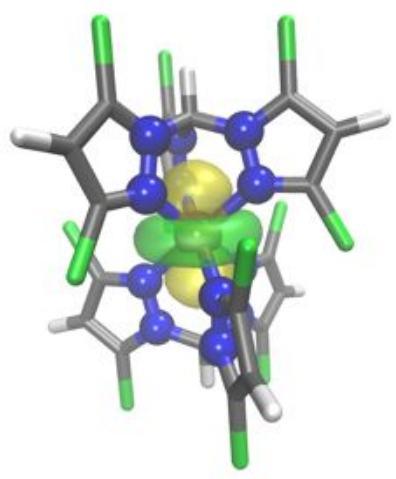<br>0 cm^{-1}

Tpm-3,5-Br
Δ_{Oh}=16492cm^{-1}

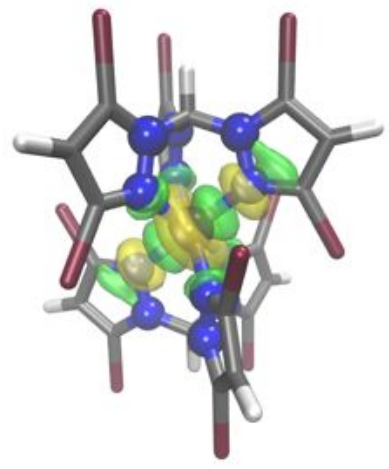

19822 cm^{-1}

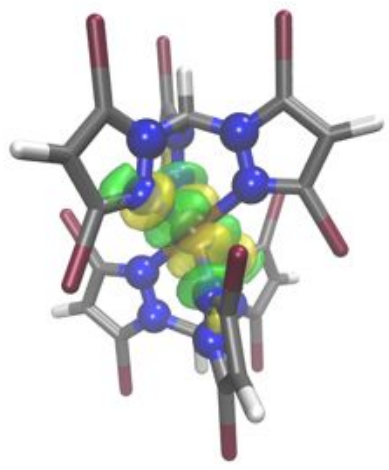

19692 cm^{-1}

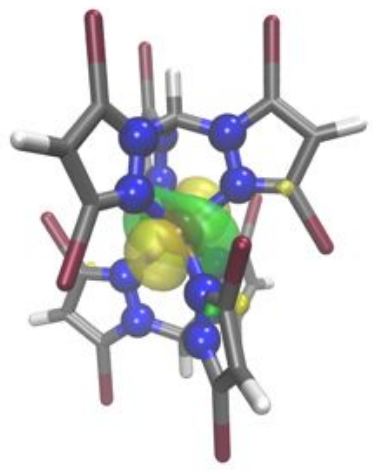

3200 cm^{-1}

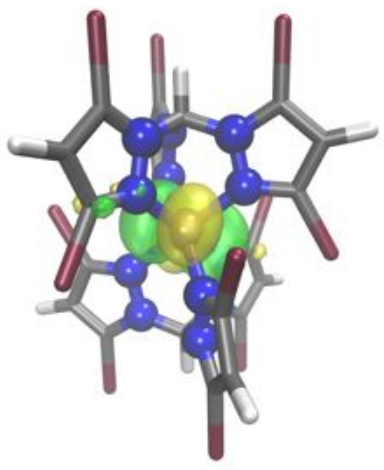

3196 cm^{-1}

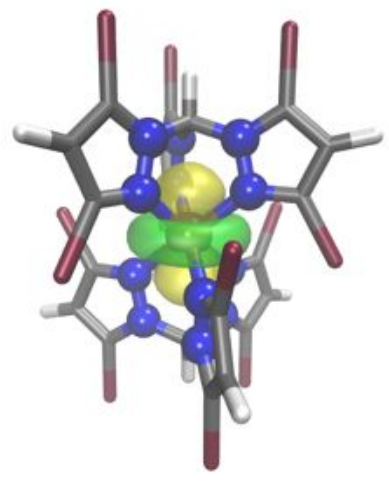

0 cm^{-1}

Tpm-3,5-I
Δ_{Oh}=16803cm^{-1}

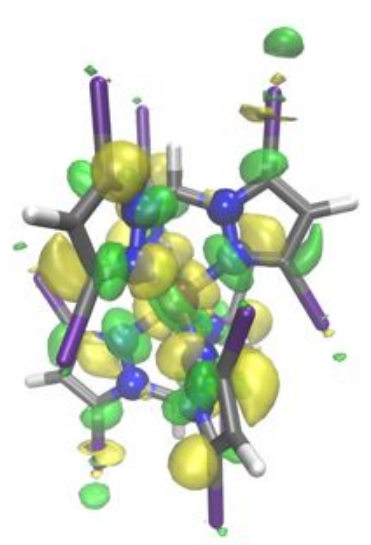

20167 cm^{-1}

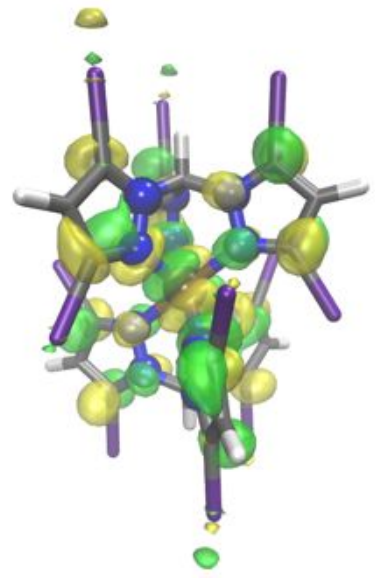

20020 cm^{-1}

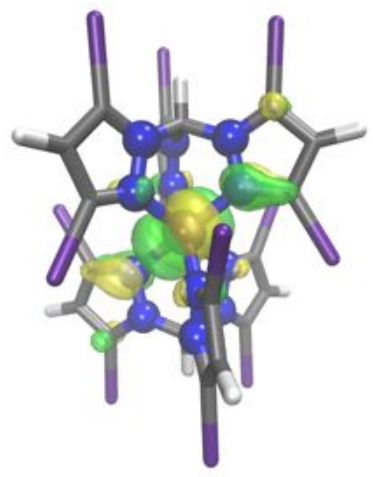

3217 cm^{-1}

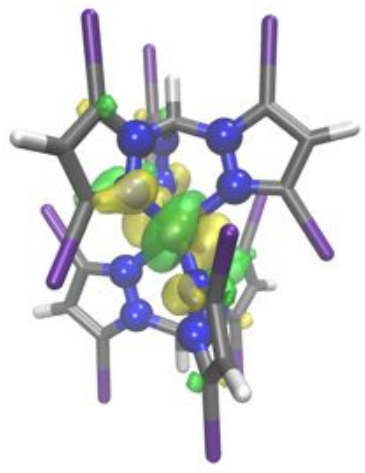

3214 cm^{-1}

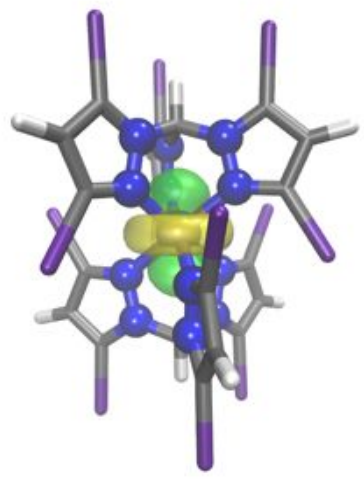

0 cm^{-1}

All energies are in cm^{-1}, corresponding to the NEVPT2/AILFT analysis of the electronic structure of the fully optimized low-spin (S=1) systems. Δ_{Oh} estimated as the energy difference between the first unoccupied d-MO and the last occupied one.

S3. Correlations for the $[Cr(Tpm^{4\text{-}R})_2]^{2+}$ systems with the R_x, I_x σ_p and σ_m Hammett parameters as well as spin-state energy gap, thermochemical quantities and computed $T_{1/2}$. Table includes also the average Cr-N bond length in both spin-states.

-R	Rx	Ix	σ_m	σ_p	ΔE	ΔH	ΔS	$T_{1/2}$	d(Cr-N)^{HS}	d(Cr-N)^{LS}
H	0.0000	0.0000	0.00	0.00	1.27	2.05	9.127	224	2.2171	2.0449
CH_3	-0.0106	-0.0010	-0.07	-0.17	1.64	2.15	8.953	240	2.2171	2.0497
NH_2	-0.0412	0.0020	-0.16	-0.66	1.22	1.72	6.527	264	2.2009	2.0481
CF_3	0.0125	0.0066	0.43	0.54	1.09	1.39	9.118	153	2.2193	2.0539
Cl	-0.0041	0.0056	0.37	0.23	1.27	1.55	8.905	174	2.2160	2.0530
COOH	0.0217	0.0054	0.37	0.45	0.87	1.18	8.841	133	2.2175	2.0521
F	-0.0173	0.0080	0.34	0.06	1.44	1.73	8.839	196	2.2142	2.0532
SMe	-0.0193	0.0035	0.15	0.00	1.77	2.45	8.496	289	2.2141	2.0417
CN	0.0147	0.0064	0.56	0.66	-0.04	0.76	8.445	90	2.2221	2.0487
NO_2	0.0253	0.0110	0.71	0.78	0.78	1.06	8.690	122	2.2215	2.0562
OCH_3	-0.0316	0.0046	0.12	-0.27	1.85	2.23	7.686	290	2.2059	2.0480
OH	-0.0338	0.0050	0.12	-0.37	1.62	2.34	7.514	312	2.2070	2.0423

All Energies and enthalpies in $kcal \cdot mol^{-1}$, entropies in $cal \cdot K^{-1} \cdot mol^{-1}$, temperatures in K, and bond lengths in Angstroms.

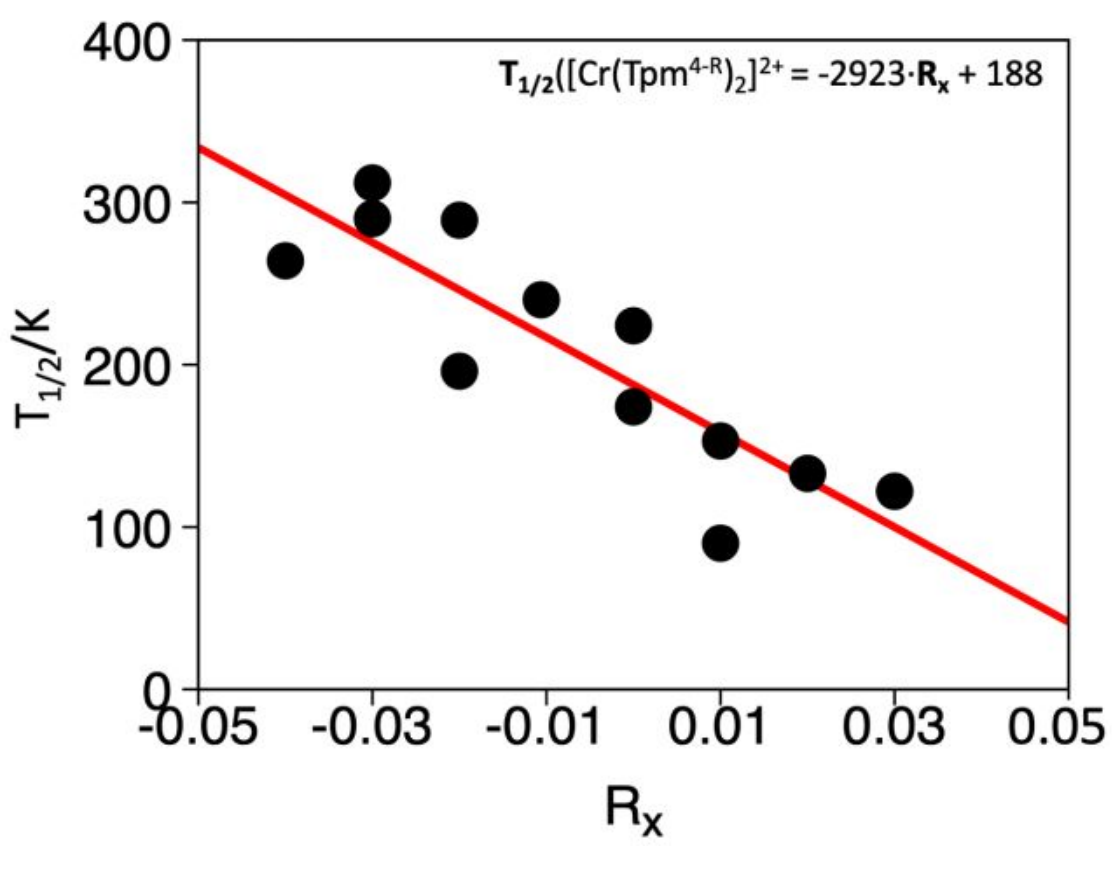

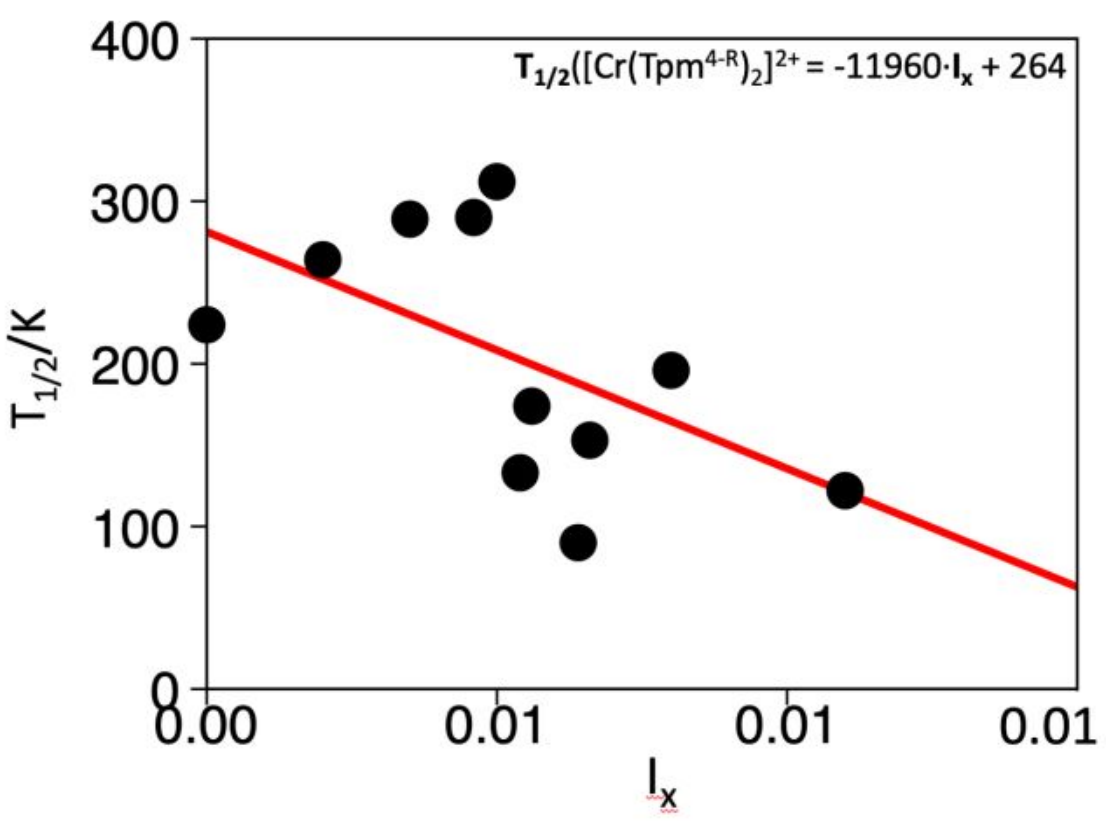

Correlation plots with the Rx (R^2 = 0.80) and Ix (R^2 = 0.29) parameters, including the linear model. As can be seen, only with the resonant parameter some trend is observed.

S4. Average value of the bite angle and donor-donor distance (d) value for the $[Cr(Tpm^{3-CH3})_2]^{2+}$, $[Cr(Tpm)_2]^{2+}$ and $[Cr(Tpm^{5-CH3})_2]^{2+}$ systems.

Low-spin:

$[Cr(Tpm^{3-CH3})_2]^{2+}$: Bite angle 85.6º and d=2.047 Å

$[Cr(Tpm)_2]^{2+}$: Bite angle 85.8º and d=2.045 Å

$[Cr(Tpm^{5-CH3})_2]^{2+}$: Bite angle 86.3º and d=2.082 Å

High-spin:

$[Cr(Tpm^{3-CH3})_2]^{2+}$: Bite angle 81.8º and d=2.208 Å

$[Cr(Tpm)_2]^{2+}$: Bite angle 81.8º and d=2.229 Å

$[Cr(Tpm^{5-CH3})_2]^{2+}$: Bite angle 82.6º and d=2.232 Å

S5. $[Cr(Tpm^{3\text{-}R})_2]^{2+}$ correlations with $\sigma_{p,}$ R_x and I_x, as well as the spin-state energy gap, thermochemical quantities and computed $T_{1/2.}$

$-R_3$	σ_p	R_x	I_x	ΔE	ΔH	ΔS	$T_{1/2}$
$-NH_2$	-0.66	-0.0412	0.0020	0.07	0.07	6.51	11
-SMe	0.00	-0.0193	0.0035	1.88	1.88	6.82	276
-F	0.06	-0.0173	0.0080	-0.34	-0.34	7.84	-43
-CH3	-0.17	-0.0106	-0.0010	2.75	2.75	8.17	336
-Cl	0.23	-0.0041	0.0056	1.05	1.05	7.94	132
-H	0.00	0.0000	0.0000	2.05	2.05	9.13	224
$-CF_3$	0.54	0.0125	0.0066	3.22	3.22	6.65	484
-CN	0.66	0.0147	0.0064	2.65	2.65	8.20	324
-COOH	0.45	0.0217	0.0054	4.29	4.29	6.45	665
$-NO_2$	0.78	0.0253	0.0110	4.27	4.27	5.98	715

All Energies and enthalpy differences in $kcal\cdot mol^{-1}$, entropy difference in $cal\cdot K^{-1}\cdot mol^{-1}$ and temperatures in K.

Correlation plots between the computed spin-state energy gap (left) and computed $T_{1/2}$ (right) against the σ_p (top) Hammett constant and Rx(bottom) parameters. Plots include the linear model and R^2 value. As can be seen, a better correlation with the R_x value is outlined. No correlation whatsoever with the I_x value is observed ($R^2 = 0.12$).

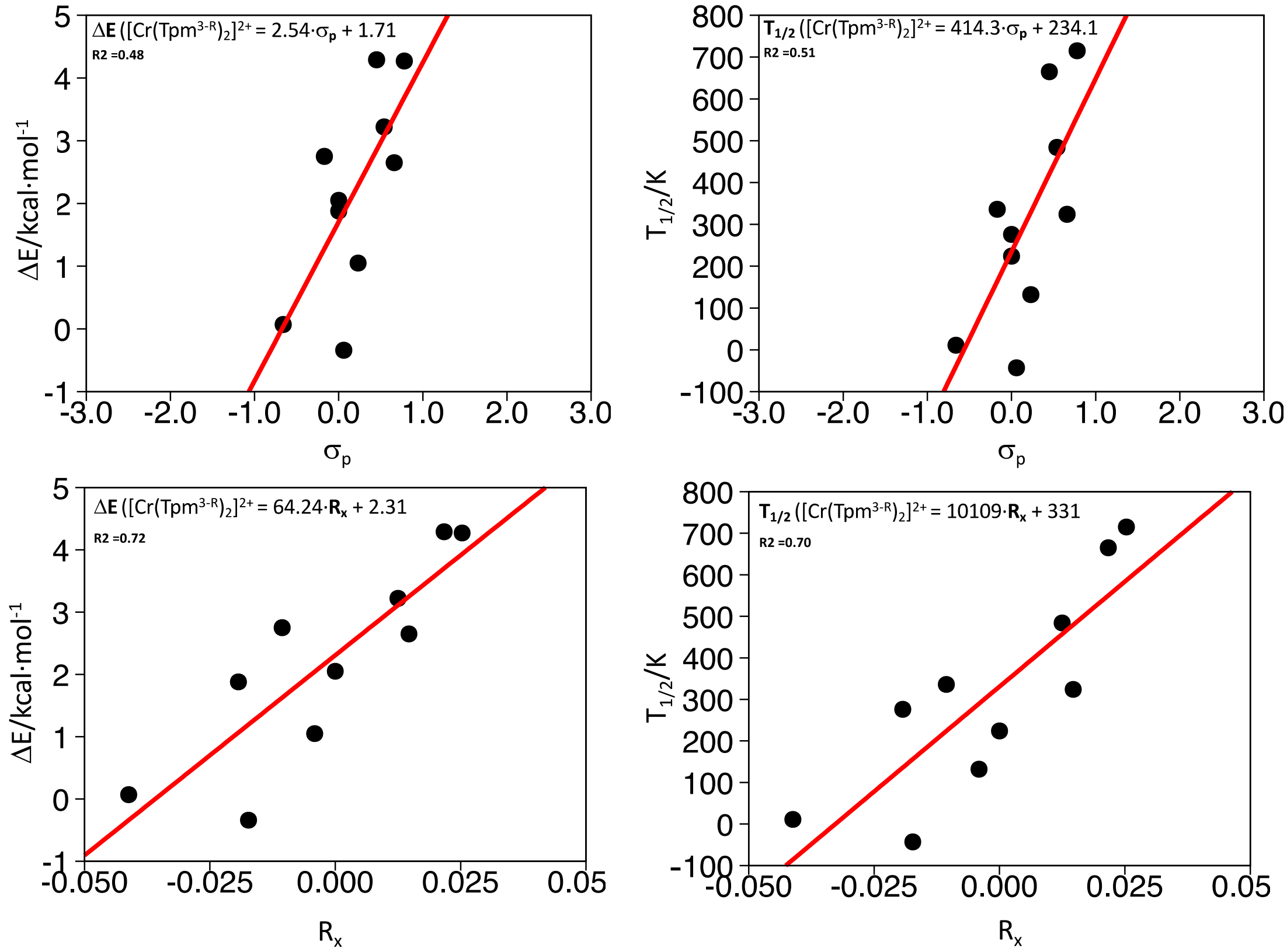

S6. Continuous Shape Measures (CShM) towards the octahedron and the trigonal prism reference shapes, as well as the path deviation (Δφ) and the generalized interconversion ($\varphi_{P\to Q}$) parameter for all systems studied in this work.

System	S(OC-6)	S(TP-6)	Δφ(%)	$\varphi_{P\to Q}$
Cr_Tpb_3-5-Me_hs	0.914	15.203	17.7	-
Cr_Tpb_3-5-Me_ls	0.035	16.263	2.9	4.4
Cr_Tpb_hs	1.189	15.485	21.9	-
Cr_Tpb_ls	0.11	15.885	5.1	7.9
Cr_Tpm_3-5-Br_hs	1.097	15.155	19.8	-
Cr_Tpm_3-5-Br_ls	0.177	15.671	6.5	10
Cr_Tpm_3-5-CF3_hs	1.036	15.145	19	-
Cr_Tpm_3-5-CF3_ls	0.094	15.668	3.8	7.3
Cr_Tpm_3-5-Cl_hs	1.198	15.126	20.8	-
Cr_Tpm_3-5-Cl_ls	0.225	15.558	7.5	11.3
Cr_Tpm_3-5-F_hs	1.652	15.12	25.3	-
Cr_Tpm_3-5-F_ls	0.36	15.316	9.6	14.2
Cr_Tpm_3-5-I_hs	0.902	15.252	17.8	-
Cr_Tpm_3-5-I_ls	0.115	15.865	5.2	8
Cr_Tpm_3-5-Me_hs	1.238	15.122	21.2	-
Cr_Tpm_3-5-Me_ls	0.207	15.487	6.8	10.8
Cr_Tpm_3-5-NH2_hs	1.658	15.173	25.6	-
Cr_Tpm_3-5-NH2_ls	0.383	15.212	9.8	14.7
Cr_Tpm_3-CF3_hs	1.62	11.705	13.1	-
Cr_Tpm_3-CF3_ls	0.284	15.241	7.8	12.7
Cr_Tpm_3-CN_hs	1.628	15.203	25.4	-
Cr_Tpm_3-CN_ls	0.243	15.533	7.8	11.7
Cr_Tpm_3-COOH_hs	1.535	12.108	13.8	-
Cr_Tpm_3-COOH_ls	0.275	15.221	7.5	12.4
Cr_Tpm_3-Cl_hs	1.575	15.175	24.8	-
Cr_Tpm_3-Cl_ls	0.264	15.488	8.2	12.2
Cr_Tpm_3-F_hs	1.615	15.218	25.3	-
Cr_Tpm_3-F_ls	0.26	15.493	8.1	12.1
Cr_Tpm_3-Me_hs	1.661	13.782	20.9	-

Cr_Tpm_3-Me_ls	0.285	15.44	8.5	12.7
Cr_Tpm_3-NH2_hs	1.768	15.312	27	-
Cr_Tpm_3-NH2_ls	0.261	15.481	8.1	12.1
Cr_Tpm_3-NO2_hs	1.486	12.228	13.8	-
Cr_Tpm_3-NO2_ls	0.262	15.068	6.7	12.1
Cr_Tpm_3-SMe_hs	1.644	12.748	17.1	-
Cr_Tpm_3-SMe_ls	0.261	15.493	8.1	12.1
Cr_Tpm_4-CF3_hs	1.676	15.115	25.5	-
Cr_Tpm_4-CF3_ls	0.291	15.425	8.6	12.8
Cr_Tpm_4-CN_hs	1.703	15.158	25.9	-
Cr_Tpm_4-CN_ls	0.283	15.454	8.5	12.6
Cr_Tpm_4-COOH_hs	1.661	15.166	25.6	-
Cr_Tpm_4-COOH_ls	0.281	15.443	8.4	12.6
Cr_Tpm_4-Cl_hs	1.635	15.071	25	-
Cr_Tpm_4-Cl_ls	0.284	15.449	8.5	12.6
Cr_Tpm_4-F_hs	1.638	14.294	22.4	-
Cr_Tpm_4-F_ls	0.28	15.454	8.4	12.6
Cr_Tpm_4-Me_hs	1.649	15.245	25.7	-
Cr_Tpm_4-Me_ls	0.27	15.471	8.2	12.3
Cr_Tpm_4-NH2_hs	1.635	12.554	16.4	-
Cr_Tpm_4-NH2_ls	0.263	15.38	7.8	12.2
Cr_Tpm_4-NO2_hs	1.698	15.008	25.4	-
Cr_Tpm_4-NO2_ls	0.298	15.408	8.7	13
Cr_Tpm_4-OCH3_hs	1.663	12.844	17.6	-
Cr_Tpm_4-OCH3_ls	0.266	15.383	7.9	12.2
Cr_Tpm_4-OH_hs	1.611	14.025	21.3	-
Cr_Tpm_4-OH_ls	0.27	15.447	8.2	12.3
Cr_Tpm_4-SMe_hs	1.648	14.397	22.9	-
Cr_Tpm_4-SMe_ls	0.253	15.476	7.9	11.9
Cr_Tpm_5-CF3_hs	0.948	15.299	18.5	-
Cr_Tpm_5-CF3_ls	0.082	15.986	4.4	6.8
Cr_Tpm_5-Cl_hs	1.279	15.089	21.5	-
Cr_Tpm_5-Cl_ls	0.249	15.51	7.9	11.8
Cr_Tpm_5-Me_hs	1.288	15.241	22.1	-
Cr_Tpm_5-Me_ls	0.207	15.086	5.5	10.8

Cr_Tpm_5-NH2_hs	1.599	12.405	15.5	-
Cr_Tpm_5-NH2_ls	0.316	15.319	8.7	13.3
Cr_Tpm_hs	1.648	15.066	25.1	-
Cr_Tpm_ls	0.266	15.484	8.2	12.2

S7. Jahn-Teller analysis of all the studied systems. Shortest Cr-N bond (in Å), longest Cr-N (in Å) bond and ratio between both values.

System	Cr-N_min	Cr-N_max	Ratio
Cr_Tpb_3-5-Me_hs	2.102300	2.451296	1.166007
Cr_Tpb_3-5-Me_ls	2.060140	2.072397	1.005950
Cr_Tpb_hs	2.088936	2.460856	1.178043
Cr_Tpb_ls	2.029624	2.048428	1.009265
Cr_Tpm_3-5-Br_hs	2.119540	2.395346	1.130125
Cr_Tpm_3-5-Br_ls	2.067356	2.071474	1.001992
Cr_Tpm_3-5-CF3_hs	2.137613	2.436325	1.139741
Cr_Tpm_3-5-CF3_ls	2.078266	2.091889	1.006555
Cr_Tpm_3-5-Cl_hs	2.115053	2.405239	1.137200
Cr_Tpm_3-5-Cl_ls	2.067807	2.070063	1.001091
Cr_Tpm_3-5-F_hs	2.105610	2.456628	1.166706
Cr_Tpm_3-5-F_ls	2.064504	2.065077	1.000278
Cr_Tpm_3-5-I_hs	2.117559	2.373757	1.120988
Cr_Tpm_3-5-I_ls	2.062909	2.068187	1.002558
Cr_Tpm_3-5-Me_hs	2.112571	2.432568	1.151473
Cr_Tpm_3-5-Me_ls	2.074483	2.079301	1.002323
Cr_Tpm_3-5-NH2_hs	2.068682	2.499253	1.208138
Cr_Tpm_3-5-NH2_ls	2.063172	2.077500	1.006945
Cr_Tpm_3-CF3_hs	2.082189	2.386146	1.145979
Cr_Tpm_3-CF3_ls	2.022458	2.049669	1.013454
Cr_Tpm_3-CN_hs	2.097028	2.478179	1.181758
Cr_Tpm_3-CN_ls	2.033741	2.055519	1.010708
Cr_Tpm_3-COOH_hs	2.072565	2.393458	1.154829
Cr_Tpm_3-COOH_ls	2.016192	2.046701	1.015132
Cr_Tpm_3-Cl_hs	2.092406	2.463315	1.177264
Cr_Tpm_3-Cl_ls	2.052725	2.055740	1.001469
Cr_Tpm_3-F_hs	2.096886	2.485848	1.185495
Cr_Tpm_3-F_ls	2.058511	2.065667	1.003476
Cr_Tpm_3-Me_hs	2.082524	2.447127	1.175077
Cr_Tpm_3-Me_ls	2.046039	2.048359	1.001134
Cr_Tpm_3-NH2_hs	2.086509	2.511427	1.203650

Cr_Tpm_3-NH2_ls	2.055028	2.060935	1.002875
Cr_Tpm_3-NO2_hs	2.086302	2.402150	1.151391
Cr_Tpm_3-NO2_ls	2.029453	2.058616	1.014370
Cr_Tpm_3-SMe_hs	2.079003	2.439460	1.173379
Cr_Tpm_3-SMe_ls	2.046615	2.050942	1.002114
Cr_Tpm_4-CF3_hs	2.095336	2.467298	1.177519
Cr_Tpm_4-CF3_ls	2.052844	2.055114	1.001106
Cr_Tpm_4-CN_hs	2.097761	2.470492	1.177681
Cr_Tpm_4-CN_ls	2.036540	2.054897	1.009013
Cr_Tpm_4-COOH_hs	2.092161	2.469197	1.180214
Cr_Tpm_4-COOH_ls	2.047035	2.056176	1.004465
Cr_Tpm_4-Cl_hs	2.092647	2.462803	1.176884
Cr_Tpm_4-Cl_ls	2.052878	2.053159	1.000137
Cr_Tpm_4-F_hs	2.089239	2.462622	1.178717
Cr_Tpm_4-F_ls	2.052928	2.053410	1.000235
Cr_Tpm_4-Me_hs	2.090969	2.470268	1.181399
Cr_Tpm_4-Me_ls	2.047765	2.052920	1.002517
Cr_Tpm_4-NH2_hs	2.077344	2.434154	1.171762
Cr_Tpm_4-NH2_ls	2.044766	2.051855	1.003467
Cr_Tpm_4-NO2_hs	2.098154	2.470609	1.177516
Cr_Tpm_4-NO2_ls	2.054823	2.057167	1.001140
Cr_Tpm_4-OCH3_hs	2.082774	2.442047	1.172497
Cr_Tpm_4-OCH3_ls	2.041675	2.052311	1.005209
Cr_Tpm_4-OH_hs	2.085888	2.456175	1.177520
Cr_Tpm_4-OH_ls	2.046465	2.052533	1.002965
Cr_Tpm_4-SMe_hs	2.085915	2.469041	1.183673
Cr_Tpm_4-SMe_ls	2.027864	2.049078	1.010461
Cr_Tpm_5-CF3_hs	2.145150	2.434974	1.135107
Cr_Tpm_5-CF3_ls	2.090168	2.095122	1.002370
Cr_Tpm_5-Cl_hs	2.116731	2.407422	1.137330
Cr_Tpm_5-Cl_ls	2.063322	2.069348	1.002920
Cr_Tpm_5-Me_hs	2.118554	2.456018	1.159290
Cr_Tpm_5-Me_ls	2.078287	2.083418	1.002469
Cr_Tpm_5-NH2_hs	2.083926	2.466893	1.183771
Cr_Tpm_5-NH2_ls	2.066695	2.074630	1.003839
Cr_Tpm_hs	2.092082	2.469203	1.180261
Cr_Tpm_ls	2.032772	2.051338	1.009133

S8. Bond length analysis for all studied systems in this work. All distances in Å.

File	Cr-N1	Cr-N2	Cr-N3	Cr-N4	Cr-N5	Cr-N6	Average
Cr_Tpb_3-5-Me_hs.xyz	2.1023	2.1023	2.1190	2.1201	2.4502	2.4513	2.2242
Cr_Tpb_3-5-Me_ls.xyz	2.0601	2.0604	2.0629	2.0631	2.0723	2.0724	2.0652
Cr_Tpb_hs.xyz	2.0889	2.0891	2.0897	2.0901	2.4583	2.4609	2.2128
Cr_Tpb_ls.xyz	2.0296	2.0298	2.0478	2.0479	2.0484	2.0484	2.0420
Cr_Tpm_3-5-Br_hs.xyz	2.1195	2.1202	2.1204	2.1207	2.3951	2.3953	2.2119
Cr_Tpm_3-5-Br_ls.xyz	2.0674	2.0674	2.0712	2.0713	2.0715	2.0715	2.0700
Cr_Tpm_3-5-CF3_hs.xyz	2.1376	2.1528	2.1748	2.1881	2.4132	2.4363	2.2505
Cr_Tpm_3-5-CF3_ls.xyz	2.0783	2.0793	2.0825	2.0871	2.0890	2.0919	2.0847
Cr_Tpm_3-5-Cl_hs.xyz	2.1151	2.1159	2.1163	2.1163	2.4046	2.4052	2.2122
Cr_Tpm_3-5-Cl_ls.xyz	2.0678	2.0679	2.0698	2.0699	2.0700	2.0701	2.0692
Cr_Tpm_3-5-F_hs.xyz	2.1056	2.1057	2.1060	2.1062	2.4562	2.4566	2.2227
Cr_Tpm_3-5-F_ls.xyz	2.0645	2.0645	2.0650	2.0650	2.0650	2.0651	2.0649
Cr_Tpm_3-5-I_hs.xyz	2.1176	2.1180	2.1185	2.1187	2.3735	2.3738	2.2034
Cr_Tpm_3-5-I_ls.xyz	2.0629	2.0629	2.0680	2.0681	2.0682	2.0682	2.0664
Cr_Tpm_3-5-Me_hs.xyz	2.1126	2.1142	2.1218	2.1225	2.4320	2.4326	2.2226
Cr_Tpm_3-5-Me_ls.xyz	2.0745	2.0751	2.0779	2.0785	2.0792	2.0793	2.0774
Cr_Tpm_3-5-NH2_hs.xyz	2.0687	2.0798	2.0893	2.0896	2.4646	2.4993	2.2152
Cr_Tpm_3-5-NH2_ls.xyz	2.0632	2.0647	2.0689	2.0759	2.0764	2.0775	2.0711
Cr_Tpm_3-CF3_hs.xyz	2.0822	2.0826	2.1156	2.1161	2.3853	2.3861	2.1947
Cr_Tpm_3-CF3_ls.xyz	2.0225	2.0243	2.0418	2.0444	2.0465	2.0497	2.0382
Cr_Tpm_3-CN_hs.xyz	2.0970	2.0973	2.0980	2.0981	2.4767	2.4782	2.2242
Cr_Tpm_3-CN_ls.xyz	2.0337	2.0338	2.0554	2.0554	2.0555	2.0555	2.0482
Cr_Tpm_3-COOH_hs.xyz	2.0726	2.0727	2.1069	2.1069	2.3928	2.3935	2.1909
Cr_Tpm_3-COOH_ls.xyz	2.0162	2.0165	2.0448	2.0451	2.0458	2.0467	2.0359
Cr_Tpm_3-Cl_hs.xyz	2.0924	2.0926	2.0932	2.0935	2.4612	2.4633	2.2160
Cr_Tpm_3-Cl_ls.xyz	2.0527	2.0528	2.0555	2.0556	2.0556	2.0557	2.0547
Cr_Tpm_3-F_hs.xyz	2.0969	2.0969	2.0974	2.0976	2.4832	2.4858	2.2263
Cr_Tpm_3-F_ls.xyz	2.0585	2.0585	2.0653	2.0653	2.0656	2.0657	2.0632
Cr_Tpm_3-Me_hs.xyz	2.0825	2.0827	2.0936	2.0940	2.4427	2.4471	2.2071
Cr_Tpm_3-Me_ls.xyz	2.0460	2.0464	2.0469	2.0469	2.0482	2.0484	2.0471
Cr_Tpm_3-NH2_hs.xyz	2.0865	2.0874	2.0878	2.0888	2.5043	2.5114	2.2277
Cr_Tpm_3-NH2_ls.xyz	2.0550	2.0551	2.0578	2.0584	2.0604	2.0609	2.0580
Cr_Tpm_3-NO2_hs.xyz	2.0863	2.0869	2.1108	2.1121	2.4021	2.4021	2.2001
Cr_Tpm_3-NO2_ls.xyz	2.0295	2.0316	2.0356	2.0386	2.0573	2.0586	2.0419
Cr_Tpm_3-SMe_hs.xyz	2.0790	2.0804	2.0955	2.0957	2.4387	2.4395	2.2048
Cr_Tpm_3-SMe_ls.xyz	2.0466	2.0468	2.0503	2.0505	2.0507	2.0509	2.0493
Cr_Tpm_4-CF3_hs.xyz	2.0953	2.0954	2.0961	2.0964	2.4651	2.4673	2.2193
Cr_Tpm_4-CF3_ls.xyz	2.0528	2.0530	2.0535	2.0540	2.0547	2.0551	2.0539

Cr_Tpm_4-CN_hs.xyz	2.0978	2.0983	2.0983	2.0985	2.4693	2.4705	2.2221
Cr_Tpm_4-CN_ls.xyz	2.0365	2.0367	2.0546	2.0546	2.0547	2.0549	2.0487
Cr_Tpm_4-COOH_hs.xyz	2.0922	2.0937	2.0951	2.0957	2.4594	2.4692	2.2175
Cr_Tpm_4-COOH_ls.xyz	2.0470	2.0471	2.0530	2.0534	2.0558	2.0562	2.0521
Cr_Tpm_4-Cl_hs.xyz	2.0926	2.0927	2.0937	2.0939	2.4601	2.4628	2.2160
Cr_Tpm_4-Cl_ls.xyz	2.0529	2.0529	2.0530	2.0531	2.0531	2.0532	2.0530
Cr_Tpm_4-F_hs.xyz	2.0892	2.0896	2.0941	2.0947	2.4547	2.4626	2.2142
Cr_Tpm_4-F_ls.xyz	2.0529	2.0529	2.0531	2.0532	2.0533	2.0534	2.0532
Cr_Tpm_4-Me_hs.xyz	2.0910	2.0912	2.0912	2.0913	2.4678	2.4703	2.2171
Cr_Tpm_4-Me_ls.xyz	2.0478	2.0478	2.0485	2.0486	2.0527	2.0529	2.0497
Cr_Tpm_4-NH2_hs.xyz	2.0773	2.0813	2.0942	2.0944	2.4241	2.4342	2.2009
Cr_Tpm_4-NH2_ls.xyz	2.0448	2.0461	2.0480	2.0481	2.0498	2.0519	2.0481
Cr_Tpm_4-NO2_hs.xyz	2.0982	2.0987	2.0988	2.0994	2.4634	2.4706	2.2215
Cr_Tpm_4-NO2_ls.xyz	2.0548	2.0553	2.0564	2.0566	2.0569	2.0572	2.0562
Cr_Tpm_4-OCH3_hs.xyz	2.0828	2.0834	2.0928	2.0969	2.4377	2.4420	2.2059
Cr_Tpm_4-OCH3_ls.xyz	2.0417	2.0422	2.0491	2.0510	2.0517	2.0523	2.0480
Cr_Tpm_4-OH_hs.xyz	2.0859	2.0877	2.0903	2.0949	2.4390	2.4562	2.2090
Cr_Tpm_4-OH_ls.xyz	2.0465	2.0468	2.0468	2.0471	2.0521	2.0525	2.0486
Cr_Tpm_4-SMe_hs.xyz	2.0859	2.0860	2.0907	2.0916	2.4611	2.4690	2.2141
Cr_Tpm_4-SMe_ls.xyz	2.0279	2.0284	2.0480	2.0484	2.0485	2.0491	2.0417
Cr_Tpm_5-CF3_hs.xyz	2.1452	2.1517	2.1540	2.1578	2.4323	2.4350	2.2460
Cr_Tpm_5-CF3_ls.xyz	2.0902	2.0913	2.0919	2.0931	2.0946	2.0951	2.0927
Cr_Tpm_5-Cl_hs.xyz	2.1167	2.1172	2.1176	2.1176	2.4062	2.4074	2.2138
Cr_Tpm_5-Cl_ls.xyz	2.0633	2.0636	2.0689	2.0690	2.0691	2.0693	2.0672
Cr_Tpm_5-Me_hs.xyz	2.1186	2.1202	2.1203	2.1206	2.4503	2.4560	2.2310
Cr_Tpm_5-Me_ls.xyz	2.0783	2.0784	2.0828	2.0828	2.0831	2.0834	2.0815
Cr_Tpm_5-NH2_hs.xyz	2.0839	2.0883	2.0902	2.0913	2.4148	2.4669	2.2059
Cr_Tpm_5-NH2_ls.xyz	2.0667	2.0670	2.0671	2.0675	2.0746	2.0746	2.0696
Cr_Tpm_hs.xyz	2.0921	2.0926	2.0929	2.0934	2.4627	2.4692	2.2171
Cr_Tpm_ls.xyz	2.0328	2.0333	2.0505	2.0505	2.0510	2.0513	2.0449

S9. Computed spin-crossover transition temperatures ($T_{1/2}$, in K) for selected members of the $[Cr(Tpm^{5\text{-}R})_2]^{2+}$series obtained from isolated-molecule calculations and from models including explicit BF_4- and ClO_4- counterions. The counterions were placed according to the crystallographic available data, but free optimization was allowed, to evaluate the influence of ion-pairing effects on the computed spin-state energetics. Negative values indicate a high-spin ground state.

R_5	r_L_Tpm	$[Cr(Tpm)_2]^{2+}$	$[Cr(Tpm)_2](BF_4)_2$	$[Cr(Tpm)_2](ClO_4)_2$
H	1.2	224	310	307
CF_3	2.79	-271	-157	-434
Cl	1.82	66	58	-71
